# CGRRF1, a growth suppressor, regulates EGFR ubiquitination in breast cancer

**DOI:** 10.1186/s13058-019-1212-2

**Published:** 2019-12-04

**Authors:** Yu-Ju Lee, Shiuh-Rong Ho, Joshua D. Graves, Yang Xiao, Shixia Huang, Weei-Chin Lin

**Affiliations:** 1 Section of Hematology/Oncology, Department of Medicine, Baylor College of Medicine, One Baylor Plaza, MS: BCM187, Houston, TX 77030 USA; 2 Interdepartmental Program in Translational Biology and Molecular Medicine, Baylor College of Medicine, Houston, TX 77030 USA; 3 Integrative Molecular and Biomedical Sciences Graduate Program, Baylor College of Medicine, Houston, TX 77030 USA; 4 Department of Molecular and Cellular Biology, Baylor College of Medicine, Houston, TX 77030 USA

**Keywords:** CGRRF1, Tumor suppressor, EGFR, K48-linked ubiquitination, Breast cancer, Promoter hypermethylation

## Abstract

**Background:**

CGRRF1 is a growth suppressor and consists of a transmembrane domain and a RING-finger domain. It functions as a RING domain E3 ubiquitin ligase involved in endoplasmic reticulum-associated degradation. The expression of CGRRF1 is decreased in cancer tissues; however, the role of CGRRF1 in breast cancer and the mechanism(s) of its growth suppressor function remain to be elucidated.

**Methods:**

To investigate whether CGRRF1 inhibits the growth of breast cancer, we performed MTT assays and a xenograft experiment. Tumors harvested from mice were further analyzed by reverse phase protein array (RPPA) analysis to identify potential substrate(s) of CGRRF1. Co-immunoprecipitation assay was used to verify the interaction between CGRRF1 and its substrate, followed by in vivo ubiquitination assays. Western blot, subcellular fractionation, and reverse transcription quantitative polymerase chain reaction (qRT-PCR) were performed to understand the mechanism of CGRRF1 action in breast cancer. Publicly available breast cancer datasets were analyzed to examine the association between CGRRF1 and breast cancer.

**Results:**

We show that CGRRF1 inhibits the growth of breast cancer in vitro and in vivo, and the RING-finger domain is important for its growth-inhibitory activity. To elucidate the mechanism of CGRRF1, we identified EGFR as a new substrate of CGRRF1. CGRRF1 ubiquitinates EGFR through K48-linked ubiquitination, which leads to proteasome degradation. In addition to regulating the stability of EGFR, knockout of CGRRF1 enhances AKT phosphorylation after EGF stimulation. By analyzing the breast cancer database, we found that patients with low CGRRF1 expression have shorter survival. As compared to normal breast tissues, the mRNA levels of CGRRF1 are lower in breast carcinomas, especially in HER2-positive and basal-like breast cancers. We further noticed that *CGRRF1* promoter methylation is increased in breast cancer as compared to that in normal breast tissue, suggesting that CGRRF1 is epigenetically modified in breast cancer. Treatment of 5-azactidine and panobinostat restored CGRRF1 expression, supporting that the promoter of *CGRRF1* is epigenetically modified in breast cancer. Since 5-azactidine and panobinostat can increase CGRRF1 expression, they might be potential therapies for breast cancer treatment.

**Conclusion:**

We demonstrated a tumor-suppressive function of CGRRF1 in breast cancer and identified EGFR as its target.

## Background

Breast cancer is the second leading cause of cancer death in the USA, and about one in eight women will develop breast cancer during their lifetime according to SEER Cancer Statistics Review (1975–2015). The death rate of breast cancer has been decreasing since 1989 because of earlier diagnosis and improved therapy; however, there is no specific target therapy for estrogen receptor-negative, progesterone receptor-negative, and HER2-negative, i.e., triple-negative breast cancer (TNBC). TNBC is an aggressive cancer with a high rate of recurrence, and the 5-year relative survival of metastatic breast cancer is still low. Drug resistance is another issue in recurrence and progression of breast cancer. In order to develop more potent and effective treatments for breast cancer patients, we need to understand the molecular mechanisms underlying tumorigenesis and progression of breast cancer.

CGRRF1 was first identified as a p53-responsive gene, and overexpression of rat CGRRF1 inhibited colony formation of colon carcinoma, ovarian carcinoma, and glioblastoma cell lines [1, 2]. Knockdown of p53 decreases the transcription of CGRRF1; on the other hand, doxorubicin treatment induces the expression of CGRRF1 [2]. Another study shows that the expression of CGRRF1 is also controlled by RBL2/p130. Reintroduction of wild-type RBL2/p130 into Burkitt’s lymphoma cell lines, which carry a mutated nonfunctional form of RBL2/p130, upregulated the expression of CGRRF1 and regained growth control [3]. In endometrial cancer cell lines, overexpression of CGRRF1 suppresses cell proliferation and knockdown of CGRRF1 enhances cell growth. Besides, overexpression of CGRRF1 promotes metformin-induced G1 arrest and decreases phosphorylation of S6 ribosomal protein [2]. Furthermore, several studies reported that the mRNA level of CGRRF1 is downregulated in tumor tissues including testicular germ cell tumor, endometrial tumor, and colorectal cancer [2, 4]. These findings suggest that CGRRF1 is a growth suppressor and might be involved in the development and/or progression of cancer. However, the mechanism of CGRRF1 growth suppressor function remains unclear. Its role in breast cancer is also unexplored.

CGRRF1 consists of an N-terminal transmembrane domain and a C-terminal RING-finger domain. Immunofluorescence staining has shown that CGRRF1 colocalizes with endoplasmic reticulum (ER)-associated proteins, protein disulfide isomerase (PDI) and calnexin, indicating that CGRRF1 localizes to the ER [5, 6]. The RING-finger domain of CGRRF1 contains a typical C3HC4 motif which coordinates two zinc ions [1]. Many proteins containing a RING-finger domain function as E3 ubiquitin ligases that transfer ubiquitin from a ubiquitin-conjugating enzyme to their substrate proteins. ER-associated degradation (ERAD) is a mechanism that targets misfolded proteins in the ER for ubiquitination and subsequent proteasomal degradation. The main feature of the RING-type ubiquitin ligase (E3s) involved in ERAD is proteins with transmembrane domains and RING-finger domain, and CGRRF1 has both domains. Besides, ER stress inducers thapsigargin and tunicamycin upregulate the expression of CGRRF1 and overexpression of ATF6 induces the transcription of CGRRF1 [5], suggesting the possibility that CGRRF1 acts as an ERAD ligase. Although an in vitro auto-ubiquitination assay using UbcH5c as the ubiquitin-conjugating enzyme (E2) failed to demonstrate an E3 ubiquitin ligase activity for CGRRF1 [5]; another study demonstrated that CGRRF1 targets Evi (Wls/CPR177), which regulates Wnt protein secretion, for ERAD using UBE2J2 as E2 [4].

In this study, we demonstrate that CGRRF1 suppresses the growth of breast cancer and the RING-finger domain is involved in its growth-inhibitory activity. From xenograft experiments, we noticed that EGFR expression is lower in CGRRF1-overexpressing xenografts, and similar results have been confirmed in our stable CGRRF1 overexpression cell lines. On the contrary, EGFR expression is enhanced in CGRRF1 knockout or knockdown cells. We further demonstrated that CGRRF1 interacts with EGFR and ubiquitinates EGFR through K48-linked ubiquitination, which leads to proteasome degradation. We analyzed the correlation between CGRRF1 and breast cancer patients. CGRRF1 expression is lower in breast carcinoma as compared to normal breast tissue, and patients with lower CGRRF1 expression in their tumors have a worse outcome. Its downregulation is associated with *CGRRF1* promoter hypermethylation in breast cancer. We also show that CGRRF1 downregulation in breast cancer cells can be reversed by a hypomethylating agent or a histone deacetylase inhibitor, supporting an epigenetic mechanism for its downregulation in breast cancer.

## Methods

### Cell culture, transfection, and treatment

HEK293T, Lenti-X 293T, MCF7, MDA-MB-231, MDA-MB-468, and SKBR3 cells were maintained in DMEM supplemented with 10% FBS, penicillin (50 IU/ml), and streptomycin (50 μg/ml). T47D, BT-549, and HCC70 cells were maintained in RPMI supplemented with 10% FBS, penicillin (50 IU/ml), and streptomycin (50 μg/ml). U2OS cells were maintained in McCoy’s 5A supplemented with 10% FBS, penicillin (50 IU/ml), and streptomycin (50 μg/ml). Doxycycline-inducible cell lines were maintained in DMEM supplemented with 10% tetracycline-free FBS, penicillin (50 IU/ml), streptomycin (50 μg/ml), and G418 (500 μg/ml) (VWR International). All cells were grown in a humidified incubator at 37 °C with 5% CO_2_ and 95% air. Transfection was performed with a standard polyethylenimine method or PolyJet™ in vitro DNA transfection reagent (SignaGen). After transfection, cells were incubated for 48–72 h before analysis. Cells were treated with cycloheximide (Calbiochem), EGF (Fisher), MG132 (Calbiochem), panobinostat (Selleckchem), or 5-azacitidine (Sigma) with indicated concentrations and for the time points as described in each experiment.

### Generation of CGRRF1 construct

Human CGRRF1 was amplified from pDNR-LIB-CGRRF1 (purchased from Biosystems, Clone 4245551) using the primers 5′-CTCGGATCCATGGCTGCGGTGTTTCTG-3′ and 5′-CTCGAATTCTCAAAGAGTCTTCGGTTTG-3′. The PCR product was digested with *Bam*HI/*Eco*RI and then cloned to the *Bam*HI/*Eco*RI-digested pCMV-Tag2B vector. To generate FLAG-tagged RING domain mutant CGRRF1 construct (C274A/C277A), we used QuikChange II site-directed mutagenesis kits (Agilent Technologies). The primers for site-directed mutagenesis are 5′-GGAAGAGAACAGCAAGGACGCTGTTGTTGCCCAGAATGGGACTGTGAAC-3′ and 5′-GTTCACAGTCCCATTCTGGGCAACAACAGCGTCCTTGCTGTTCTCTTCC-3′. The mutation was verified by sequencing. To generate Myc-tagged wild-type and mutant (C274A/C277A) CGRRF1, FLAG-tagged wild-type and mutant CGRRF1 were digested with *Bam*HI/*Eco*RI and then subcloned to the *Bam*HI/*Eco*RI-digested pCMV-Tag3B vector. To generate constructs for virus infection, FLAG-tagged wild-type and mutant CGRRF1 were digested with *Nhe*I/*Xho*I and then subcloned to the *Nhe*I/*Xho*I-digested pLenti4.1 vector. The sequences of all constructs were verified by sequencing in the BCM DNA sequencing core facility. The lentiviral pGIPZ CGRRF1 shRNAs #1 and #2 (V3LHS_640076 and V3LHS_216320) were purchased from Thermo Fisher Scientific. The GIPZ nonsilencing lentiviral shRNA control plasmid was purchased from Open Biosystems.

### Generation of stable CGRRF1 overexpression and knockdown cell lines

Lentiviruses were produced in Lenti-X 293T cell lines with pLenti-CGRRF1 or shCGRRF1, with psPAX2 and pMD2.G. Lentiviruses were collected and added to cells cultured with growing medium and 8 μg/ml PolyBrene (Sigma-Aldrich). The medium was changed the next day, and the cells were placed into puromycin (Gibco) selection at 72 h post-infection.

### Knockout cell line generation

Three pairs of guide oligos which target exon 1 (sgCGRRF1#1F 5′-CACCGCCGCTTTTCTACATCGCGG-3′, sgCGRRF1#1R 5′-AAACCCGCGATGTAGAAAAGCGGC-3′; sgCGRRF1#2F 5′-CACCCGTGACCACCGGCCTGGTAT-3′, sgCGRRF1#2R 5′-AAACATACCAGGCCGGTGGTCACG-3′) or exon 3 (sgCGRRF1#3F 5′-CACCAAGGTCAATAGCGCTACCAA-3′, sgCGRRF1#3R 5′-AAACTTGGTAGCGCTATTGACCTT-3′) of CGRRF1 were synthesized, annealed, and cloned into the *Bsm*BI-digested pLentiCRISPR V2 vector. The empty or cloned constructs were transfected into MCF7 and MDA-MB-231. Seventy-two hours post transfection, cells were placed into puromycin selection and plated in serial dilutions to facilitate single-cell colony formation. Ten to fourteen days post plating, single-cell colonies were isolated using cloning cylinders and subsequently amplified. Clones were screened for the knockout of CGRRF1 by western blot.

### Generation of CGRRF1 doxycycline-inducible cell lines

Inducible constructs were created by PCR amplifying respective CGRRF1 sequences out of Tag2B parental vectors using Tag2B-AttB-F (5′-GGGGACAAGTTTGTACAAAAAAGCAGGCTTCACCATGGATTACAAGGATGACGACG-3′) and Tag2B-AttB-R (5′-GGGGACCACTTTGTACAAGAAAGCTGGGTcGGGTACACTTACCTGGTACCTTAAT-3′) primers. The PCR product was gel-purified and subsequently cloned into pDONR221 using the standard gateway BP clonase reaction (Invitrogen: BP clonase Cat# 12536017). After sequence validation, pDONR221 CGRRF1 clones were cloned into pInducer20 using the standard gateway LR reaction (Invitrogen: LR clonase Cat# 11791020). The empty or CGRRF1 cloned constructs were transfected into MDA-MB-231. Seventy-two hours post transfection, cells were placed into G418 (VWR International) selection.

### Generation of stable EGFR-overexpressing cell line

The wild-type CGRRF1 doxycycline-inducible MDA-MB-231 cell line was transfected with pcDNA6 or pcDNA6A-EGFR-WT (addgene #42665) construct. Cells were placed into Blasticidin (Gibco) selection (30 μg/ml) 72 h after transfection.

### MTT assay

Cells were seeded to a 96-well plate with 500–1000 cells per well. The blank control group was without cells. MTT (0.5 mg/ml) was added to the plate at indicated time points and incubated for 2 h in an incubator. Following MTT incubation, medium and MTT were removed, and then added 100% DMSO to dissolve the crystals. The optical density (OD) was measured with a spectrophotometer at 570/630 nm.

### Colony formation assay

Cells were seeded in six-well plates at low density (200–500 cells per well) and cultured until colonies of cells appeared. The plates were then washed with PBS, fixed with 4% formaldehyde for 30 min, and then stained with 5% crystal violet. Plates were scanned and counted for colonies (each colony contained more than 50 cells).

### Pulldown assay, co-immunoprecipitation, and western blot analysis

To prepare samples for immunoprecipitation, cells were harvested 48–72 h after transfection in TGH lysis buffer (1% Triton X-100, 10% glycerol, 50 mM HEPES at pH 7.4, with 1 mM Na_3_VO_4_, sodium pyrophosphate, NaF, protease inhibitor cocktails (PIC) I, and PIC II). The lysates were then sonicated and clarified by 10-min centrifugation at 14,000 rpm. Pulldown was performed by incubating cell lysates with GFP-Trap agarose (Chromotek) or anti-FLAG M2 monoclonal antibody-conjugated agarose beads (Sigma) overnight at 4 °C. For co-immunoprecipitation, cells were lysed in TGH lysis buffer, sonicated, and clarified by 10-min centrifugation at 14,000 rpm. Control IgG or EGFR (D-8) (Santa Cruz) or CGRRF1 (Sigma, SAB1407038) antibody was pre-incubated with protein G beads (Pierce) for 2 h, then incubated with cell lysates overnight at 4 °C. Beads were washed three times with TNN (50 mM Tris at pH 7.5, 100 mM NaCl, 5 mM EDTA at pH 8.0, and 0.5% NP-40) lysis buffer, once with 0.5 M LiCl buffer, and two more times with TNN lysis buffer. Immunoprecipitants and input protein lysates were fractionated by SDS-PAGE and analyzed by western blot using indicated antibodies. To prepare samples for western blot, cells were lysed in SDS lysis buffer (60 mM Tris at pH 6.8, 1% SDS), boiled for 5 min, and then sonicated. Lysates were fractionated by SDS-PAGE, electrotransferred to an Immobilon-P membrane, and then probed with indicated primary antibodies. Some membranes were then incubated with an HRP-conjugated secondary antibody, and the signals were captured with X-ray films using enhanced chemiluminescence (ECL). Some membranes were incubated with near-infrared fluorescent secondary antibody (anti-mouse IgG (H+L) (DyLight™ 680 conjugate) and anti-rabbit IgG (H+L) (DyLight™ 800 4X PEG conjugate), purchased from Cell Signaling), and the signals were detected with Odyssey CLx infrared imaging system (LI-COR). The antibodies specific to GAPDH, HA (Y11), GFP (B-2), c-Myc (A-14), and ERα (HC-20), were purchased from Santa Cruz Biotechnology. β-Actin, phospho-EGFR (Y1068) (D7A5), EGFR (D38B1), phospho-AKT (Ser473) (D9E), phospho-AKT (T308) (D25E6), AKT (40D4), HSP90 (C45G5), phospho-Erk1/2 (Thr202/Tyr204) (197G2), c-Myc (D84C12), Aurora A (D3E4Q), and Cyclin D1 (92G2) antibodies were purchased from Cell Signaling Technology. CGRRF1 (HPA002930), FLAG, and Vinculin antibodies and anti-FLAG-HRP (A8592) were purchased from Sigma. p84 (5E10) antibody was purchased from GeneTex. Anti-acetyl-Histone H4 (Lys16) antibody was purchased from Millipore.

### In vivo ubiquitination assay

Cells were transfected with HA-UbK48, HA-UbK0 (ubiquitin carrying no lysines, Addgene#17603), or HA-UbK63 and lysed in radioimmune precipitation assay (RIPA) buffer (50 mM Tris at pH 8.0, 150 mM NaCl, 0.5% sodium deoxycholate, 1% NP-40, and 0.1% SDS, with 1 mM Na_3_VO_4_, PIC I, and PIC II) The lysates were then sonicated and clarified by 10-min centrifugation at 14,000 rpm. Equivalent amounts of cell lysates were incubated with HA-agarose beads (Sigma) overnight at 4 °C. Beads were washed three times with RIPA buffer, once with 0.5 M LiCl buffer, and two more times with RIPA buffer. The beads were then boiled in 4X SDS sample buffer and analyzed by SDS-PAGE, followed by western blot using EGFR and HA antibodies.

### Immunofluorescence staining

Immunofluorescence staining was performed as previously described [7]. In brief, cells were fixed with 4% paraformaldehyde for 10 min followed by permeabilization with 0.5% Triton X-100 in 1× PBS for 10 min. The fixed cells were then stained with indicated primary antibodies and then with a fluorescein isothiocyanate- or Texas Red-X-conjugated secondary antibody. The nuclei were stained with Hoechst 33258. Images were captured on a Zeiss fluorescence microscope (Axio Observer inverted microscope) equipped with ApoTome.2 (Zeiss).

### Subcellular fractionation assay

Cells were harvested in hypotonic lysis buffer (20 mM Tris at pH 7.4, 10 mM NaCl, 3 mM MgCl_2_, and 0.5 mM DTT) and incubated on ice for 30 min, added NP-40 to final 0.1% before dounce homogenization, and then spun down in a microfuge at 500×*g* for 10 min. The supernatant is the cytosolic fraction, and the pellet (nuclear fraction) was dissolved in SDS lysis buffer. The nuclear and cytosolic fractions were verified by western blot using antibody specific to p84 and GAPDH, respectively.

### RNA extraction and real-time RT-PCR

RNA was extracted using TRIzol reagent (Invitrogen). Quantitative PCR was performed in triplicate on an MX3005P thermal cycler using SYBR Green dye to measure amplification and ROX as a reference dye. CGRRF1 levels were normalized with GAPDH levels, which were run in parallel with CGRRF1. The results were analyzed with MxPro 4.1 Quantitative PCR software (Stratagene). The primers used for quantitative PCR were as follows: human CGRRF1-F 5′-GCTGCGGTGTTTCTGGTAAC-3′, human CGRRF1-R 5′-TGCCAGTTGTAATTGAAGCTGA-3′; GAPDH-F 5′-TGAAGGTCGGAGTCAACGGATTTGGT-3′, GAPDH-R 5′-CATGTGGGCCATGAGGTCCACCAC-3′.

### Animal study

CGRRF1-overexpressing MDA-MB-231 cells were injected subcutaneously into both sides of the flank of 5–6-week-old NOD *scid* IL2 receptor γ chain knockout (NSG) female mice. The tumor size was measured twice per week with a caliper and calculated based on the formula *π*/6 (length × depth × width). Mice were euthanized when tumor size reached 1.5 cm, and tumors were harvested. The final tumor volume and tumor weight of the xenografts on each mouse were calculated by averaging both sides of tumors. All experiments were performed under a Baylor College of Medicine Institutional Animal Care and Use Committee (IACUC)-approved protocol, and all experiments conform to IACUC standards and ethical regulations.

### Reverse phase protein array analysis

Xenografts were lysed and sonicated in ice-cold RPPA lysis buffer (Tissue Protein Extraction Reagent with 450 mM NaCl, provided by BCM RPPA Core) supplemented with protease and phosphatase inhibitors. Lysates were spun at 14,000×*g* for 15 min at 4 °C, and the supernatants were transferred to fresh tubes. The centrifugation was repeated until the supernatants were clear. Protein concentration was determined by BCA assay (Pierce™). Lysates of 0.5 mg/ml were denatured in 2× SDS sample buffer with 2.5% 2-mercaptoethanol at 100 °C for 8 min. The RPPA was performed and analyzed as previously described [8] by the Antibody-based Proteomics Core Facility at Baylor College of Medicine. Samples were probed with 236 antibodies.

### Statistical analyses

Two-tailed *t* test was performed to evaluate the differences between experimental groups. *p* values less than 0.05 were considered statistically significant. CGRRF1 expression in the TCGA (BRCA) RNA-seq database (Illumina HiSeq) and EGFR protein levels in the TCGA (BRCA) RPPA database were extracted through the xena.ucsc.edu server. Gene expression and clinical data in the METABRIC breast cancer dataset were extracted from the https://www.synapse.org/ server. Kaplan-Meier curves of breast cancer patients in the van de Vijver database was generated using the R program. Kaplan-Meier curves in Luminal A and HER2-positive breast cancer patients, kidney renal clear cell carcinoma, kidney renal papillary cell carcinoma, and lung adenocarcinoma patients were generated using KM Plotter (auto select best cutoff, overall survival, included all database). CGRRF1 gene expression (FPKM) and promoter methylation of 76 pairs of normal breast and breast tumor tissues in the TCGA (BRCA) database was extracted through the tcgaportal.org server. *CGRRF1* promoter methylation and gene expression (RNA-seq) of breast carcinoma in the TCGA (BRCA) database were extracted through the xena.ucsc.edu server. *CGRRF1* promoter methylation (HM459) and gene expression (RNA-seq) of cervical carcinoma, adrenocortical carcinoma, sarcoma, diffused large B cell lymphoma, and lung squamous carcinoma in the TCGA provisional database were extracted through the www.cbioportal.org server.

## Results

### Knockdown of CGRRF1 promotes the growth of breast cancer cell lines

**Fig. 1:**
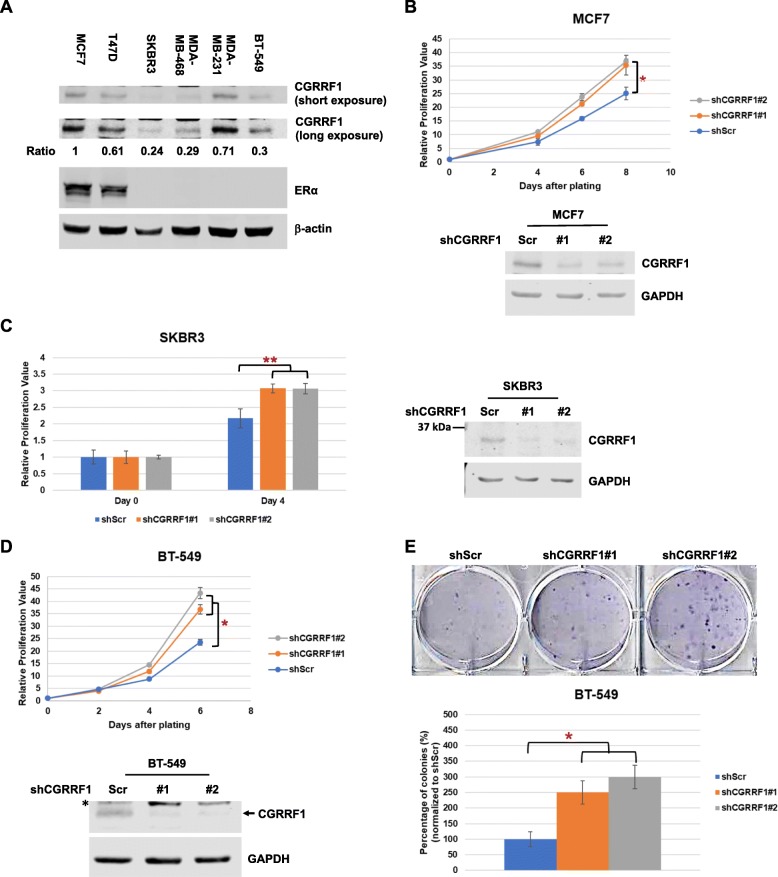
Knockdown of CGRRF1 enhances the growth of breast cancer cell lines. **a** Western blot analysis of CGRRF1 in a panel of breast cancer cell lines with different estrogen receptor status. The intensities of CGRRF1 and loading control β-actin in each lane were quantified using the Odyssey CLx infrared imaging system, and the signals of CGRRF1 were normalized by the corresponding β-actin signals and shown as relative to that of MCF7 cells. **b** The growth rate of CGRRF1-knockdown MCF7 cell lines was determined by MTT assay. Error bars represent mean ± SD (*n* = 5). *p < 0.01. Knockdown of CGRRF1 was confirmed by western blot analysis (lower panel). **c** The growth rates of CGRRF1-knockdown SKBR3 cell lines were determined by MTT assay. Error bars represent mean ± SD (*n* = 3). **p < 0.001. Knockdown of CGRRF1 was confirmed by western blot analysis (right panel). **d** The growth rates of CGRRF1-knockdown BT-549 cell lines were determined by MTT assay. Error bars represent mean ± SD (*n* = 9). *p < 0.01. Knockdown of CGRRF1 was confirmed by western blot analysis (lower panel). A nonspecific band was marked with an asterisk (*). **e** Clonogenic cell survival assay of CGRRF1-knockdown BT-549 cell lines. Error bars represent mean ± SD (*n* = 3). *p < 0.01. (A complete set of colony formation is presented in Additional file 1: Figure S1A.)

Previous studies suggest a growth repressor function for CGRRF1; however, its role in breast cancer has not been determined. We first examined the expression of CGRRF1 by western blot analysis in a panel of breast cancer cell lines (Fig. 1a). Among the cell lines that we examined, estrogen receptor-positive MCF7 and T47D cells expressed a relatively high level of CGRRF1, whereas TNBC cell lines such as MDA-MB-468 and BT-549 and a HER2-positive cell line SKBR3 had relatively lower levels of CGRRF1. To study the effect of CGRRF1 on the growth of breast cancer cell lines, we used two shRNAs (shCGRRF1#1 and shCGRRF1#2) to generate stable CGRRF1-knockdown cell lines. In MCF7, both CGRRF1-knockdown cell lines grew faster than the control cells (shScr) (Fig. 1b). Similar results were obtained in SKBR3 and BT-549 cells (Fig. 1c, d). CGRRF1 depletion in BT-549 cells also promoted the ability of cells to form colonies (Fig. 1e and Additional file 1: Figure S1A). These data demonstrate a growth suppressor function for CGRRF1 in breast cancer cells.

### Overexpression of CGRRF1 represses the growth of breast cancer cell lines

**Fig. 2:**
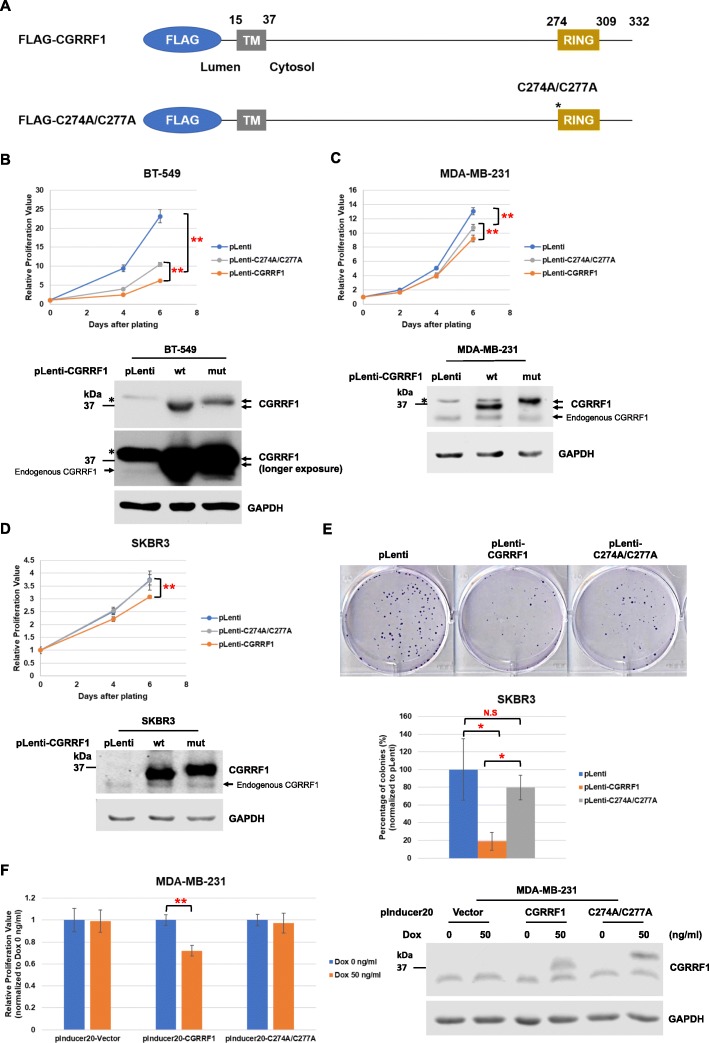
Overexpression of CGRRF1 suppresses growth of breast cancer cell lines at least in part through its RING-finger domain. **a** Wild-type and RING-domain mutant (C274A/C277A) CGRRF1 constructs used in this study. Mutation sites are indicated with an asterisk (*). **b**–**d** MTT assay was performed to examine the effect on cell proliferation by CGRRF1 in BT-549 cells (*n* = 5) (**b**), MDA-MB-231 cells (*n* = 9) (**c**), and SKBR3 cells (*n* = 5) (**d**). Error bars represent mean ± SD. **p < 0.001. Expression of CGRRF1 was validated by western blot analysis. CGRRF1 signals are indicated by arrows, and a nonspecific band was marked with an asterisk (*). **e** Clonogenic cell survival assay of CGRRF1-overexpressing SKBR3 cell lines. Error bars represent mean ± SD (*n* = 3). *p < 0.05. N.S.: not significant. A complete set is presented in Additional file 1: Figure S1B. **f** MTT assay was performed to investigate the effect of doxycycline-induced expression of CGRRF1 on the proliferation of MDA-MB-231 cells. Cells were treated with 50 ng/ml doxycycline to induce the expression of CGRRF1 for 6 days. The induction of wild-type and mutant CGRRF1 was validated by western blot analysis. Error bars represent mean ± SD (*n* = 6). **p < 0.001

Since knockdown of CGRRF1 enhanced the proliferation of breast cancer cell lines, we generated stable CGRRF1-overexpressing cell lines to study whether overexpression of CGRRF1 could inhibit cell growth. To further understand whether the RING-finger domain of CGRRF1 is involved in its function in growth regulation, we also generated a mutant construct in which two cysteine residues were mutated to alanine (C274A/C277A) to disrupt the structure of the RING-finger domain (Fig. 2a). Indeed, overexpression of wild-type CGRRF1 suppressed the proliferation of BT-549, MDA-MB-231, and SKBR3 cell lines (Fig. 2b–d). However, mutant CGRRF1 partially lost its growth-inhibitory activity. We further performed colony formation assay in SKBR3 cells. Our result showed that overexpression of wild-type but not RING-finger mutant CGRRF1 inhibited their ability to form colonies, although the size of colonies of mutant CGRRF1-overexpressing cells was smaller than control cells (Fig. 2e and Additional file 1: Figure S1B).

Since the expression of mutant CGRRF1 is usually lower than the expression of wild-type CGRRF1 in our stable cell lines (Fig. 2b, c), we performed a cycloheximide chase assay to examine the protein stability of wild-type and mutant CGRRF1. As shown in Additional file 2: Figure S2A, mutant CGRRF1 is less stable than wild-type CGRRF1. Interestingly, we noticed that the serum concentration in the media affects the expression of CGRRF1. The level of exogenously expressed CGRRF1 increased upon serum starvation (Additional file 2: Figure S2B). While the expression of mutant CGRRF1 was less than that of wild-type CGRRF1 when these stable MDA-MB-231 cells were cultured under 5% serum-containing media, the expression of mutant CGRRF1 was in fact higher under serum starvation, consistent with a shorter half-life for mutant CGRRF1 (Additional file 2: Figure S2B). To further examine the growth suppressing effect of CGRRF1, we generated CGRRF1 doxycycline-inducible MDA-MB-231 cell lines in which the expression of both wild-type and mutant CGRRF1 could be induced by doxycycline to the same levels (Fig. 2f). Indeed, wild-type CGRRF1 repressed cell proliferation; however, we did not notice significant growth inhibition in cells that expressed mutant CGRRF1. These data suggest that the RING-finger domain is important for the growth-inhibitory activity of CGRRF1.

### CGRRF1 inhibits breast cancer growth in vivo

**Fig. 3:**
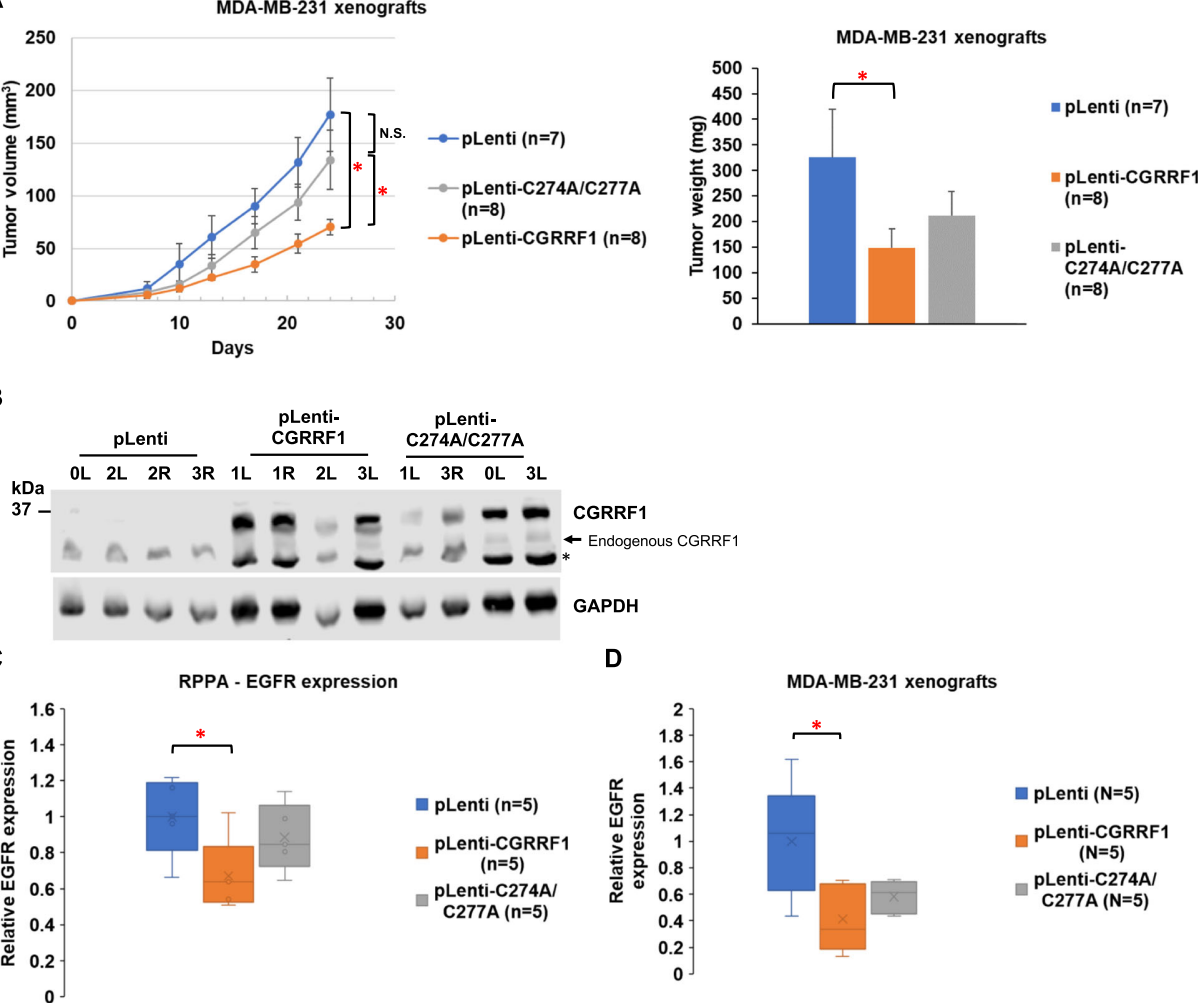
CGRRF1 inhibits the growth of breast cancer in vivo. **a** The stably transduced MDA-MB-231 cell lines as indicated were used to establish xenografts in NSG mice. Tumors were harvested on day 26. Left panel: tumor volumes; right panel: weights of xenografts. The final tumor volumes and tumor weights were calculated by averaging both sides of tumors. There was one mouse in the pLenti group which was only injected on one side. N represents the total number of tumors harvested. Error bars represent mean ± SE (*p < 0.05, N.S.: not significant). **b** The expression of CGRRF1 in xenografts was verified by western blot analysis. The asterisk (*) labels a band recognized by CGRRF1 antibody, which could be a degradation product or nonspecific staining. **c** Box plot of the levels of EGFR protein in xenografts obtained from RPPA analysis. The EGFR level was shown relative to the average of the pLenti group. *p < 0.05. **d** Box plot of the levels of EGFR in xenografts verified by western blot analysis. The EGFR level was normalized by loading control vinculin and then shown as relative to the average of pLenti xenografts (western blot is shown in Additional file 3: Figure S3C). *p < 0.05

To investigate the effect of CGRRF1 on breast tumor growth in vivo, we injected stable CGRRF1-overexpressing MDA-MB-231 cells into NOD *scid* IL2 receptor γ chain knockout (NSG) female mice. As shown in Fig. 3a, the tumor volumes and tumor weights were significantly decreased in the wild-type group (pLenti-CGRRF1) as compared to the control group (pLenti). Consistent with the data in Fig. 2c, the tumor sizes of the mutant (C274A/C277A) CGRRF1 group were between those in the control group and wild-type CGRRF1 group. The expression of wild-type and mutant CGRRF1 in these xenografts was verified by western blot analysis (Fig. 3b).

To identify the relevant target(s) of CGRRF1 in MDA-MB-231 breast cancer xenografts, the lysates of five tumors from each group were subjected to reverse phase protein array (RPPA) analysis which measured the expression of 236 proteins in triplicate from each tumor. The RPPA analysis identified 13 proteins which were expressed differentially between pLenti control and wild-type CGRRF1 at *p* < 0.05, and the changes are greater than 1.25-fold (a summary is presented as a heatmap in Additional file 3: Figure S3A). Among them, six were upregulated (14-3-3ζ/γ/ε, PDGFRβ, pS15-p53, pErk1/2 (T202/Y204), Integrin α4, and pSmad2(S465/467)), and seven were downregulated (epidermal growth factor receptor (EGFR), KLF4, p21, pBad (S136 and S155), Axl, pS1981-ATM, and IKKα) in wild-type CGRRF1-expressing xenografts. Their expressions in mutant CGRRF1-expressing xenografts fall between the pLenti control group and wild-type CGRRF1 group, but were more variable and not statistically different from the other groups. The RPPA quantitative analysis of EGFR expression in these three groups is shown in Fig. 3c. Consistent with the growth difference, the expression of Ki67 was also higher in the pLenti group and lowest in the wild-type CGRRF1 group (Additional file 3: Figure S3B), although the differences do not reach statistical significance due to small sample numbers. We also verified the RPPA result of EGFR expression using western blot analysis (Fig. 3d and Additional file 3: Figure S3C).

CGRRF1 has been demonstrated to be an E3 ubiquitin ligase of Evi and regulates its stability through ERAD [4]. Given the ER localization of CGRRF1, we suspected the proteins localized to the ER or plasma membrane would be more likely to be the direct targets of CGRRF1. Among the seven downregulated proteins in wild-type CGRRF1 xenografts, EGFR and Axl were the likely candidates. In the subsequent study, we concentrated on EGFR since we were not able to observe downregulation of Axl in cultured wild-type CGRRF1-overexpressing MDA-MB-231 cells.

### CGRRF1 interacts with EGFR

**Fig. 4:**
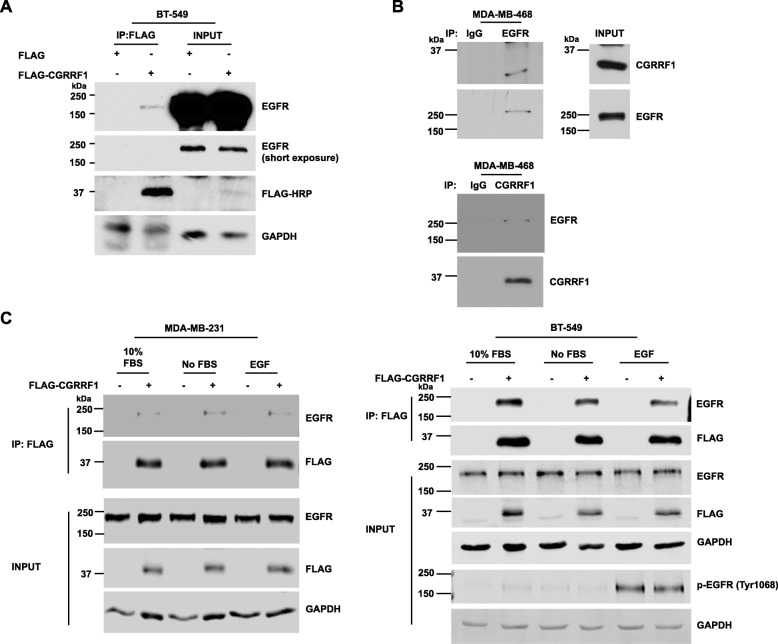
CGRRF1 interacts with epidermal growth factor receptor. **a** BT-549 cells were transfected with FLAG empty vector or FLAG-CGRRF1. Lysates were harvested 48 h after transfection and then pulled down with anti-FLAG beads, followed by immunoblotting using indicated antibodies. **b** EGFR and CGRRF1 antibodies were used to immunoprecipitate endogenous proteins in MDA-MB-468 cells, followed by immunoblotting using indicated antibodies. **c** MDA-MB-231 (left panel) and BT-549 (right panel) were transfected with FLAG-CGRRF1, serum-starved for 24 h, and then added 100 ng/ml EGF for 15 min. Cells were harvested and lysates were pulled down with anti-FLAG beads followed by immunoblotting using indicated antibodies

To examine the interaction between CGRRF1 and EGFR, HEK293T cells were transiently transfected with a plasmid that expressed GFP or GFP-tagged EGFR, and then the cell lysates were subjected to GFP-pulldown/western blot analysis. The result showed that GFP-tagged EGFR interacted with endogenous CGRRF1 (Additional file 4: Figure S4). Conversely, when FLAG-tagged CGRRF1 was transiently transfected in BT-549 cells, it interacted with endogenous EGFR (Fig. 4a). We also performed reciprocal immunoprecipitation using EGFR and CGRRF1 antibodies in MDA-MB-468 cells. As shown in Fig. 4b, CGRRF1 interacted with EGFR in MDA-MB-468 cells at their endogenous protein levels.

EGFR is a receptor tyrosine kinase which upon ligand stimulation regulates cell proliferation, survival, differentiation, migration, and angiogenesis. EGF is one of the most common ligands to activate EGFR. To study whether the interaction between CGRRF1 and EGFR is regulated by ligands, MDA-MB-231 and BT-549 cells were transfected with FLAG-CGRRF1, starved for 24 h, and then stimulated with 100 ng/ml EGF for 15 min. Cells were then harvested for FLAG-pulldown/western blot analysis. Interestingly, EGF stimulation did not affect the interaction between CGRRF1 and EGFR (Fig. 4c). Taken together, these data demonstrate that CGRRF1 can interact with EGFR in a ligand-independent manner and suggest that EGFR might be a substrate for CGRRF1 E3 ligase activity.

### CGRRF1 promotes K48-linked EGFR ubiquitination

**Fig. 5:**
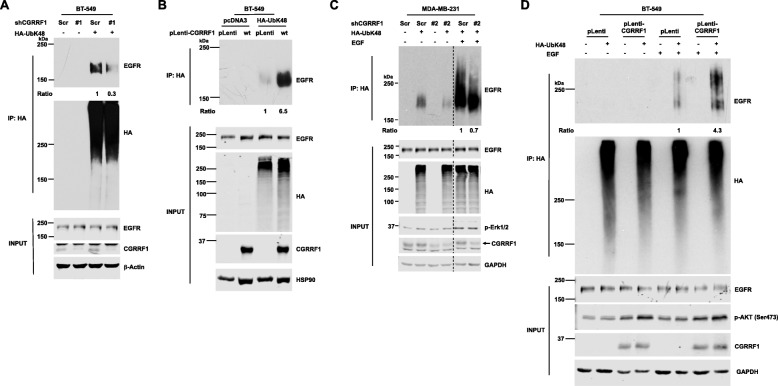
CGRRF1 ubiquitinates EGFR through K48-liked ubiquitination. **a** CGRRF1-knockdown BT-549 cell lines were transfected with pcDNA3 or HA-UbK48. Lysates were harvested 48 h after transfection and then subjected to in vivo ubiquitination assay as described in “Methods.” **b** CGRRF1-overexpressing BT-549 cell lines were transfected with pcDNA3 or HA-UbK48. Lysates were harvested 48 h after transfection and subjected to in vivo ubiquitination assay. **c** CGRRF1-knockdown MDA-MB-231 cell lines were transfected with pcDNA3 or HA-UbK48. The next day, cells were serum-starved for 24 h and then treated with 100 ng/ml EGF for 30 min. Cell lysates were harvested and in vivo ubiquitination assay was performed. The CGRRF1 signal was indicated by an arrow. **d** CGRRF1-overexpressing BT-549 cell lines were transfected with pcDNA3 or HA-UbK48. The next day, cells were serum-starved for 24 h and then treated with 100 ng/ml EGF for 5 min. Cell lysates were then harvested for in vivo ubiquitination assay

Since we have identified EGFR as a CGRRF1-interacting protein, we next performed in vivo ubiquitination assay to investigate whether CGRRF1 regulates EGFR ubiquitination. Under normal cell growing condition, we found that knockdown of CGRRF1 decreased lysine 48 residue (K48) linkage-specific ubiquitination of EGFR (Fig. 5a). In contrast, overexpression of wild-type CGRRF1 promoted K48-linked ubiquitination of EGFR (Fig. 5b).

EGF-induced EGFR ubiquitination is important for controlling EGFR internalization, trafficking, and degradation. With EGF stimulation, we observed that knockdown of CGRRF1 diminished K48-linked EGFR ubiquitination in both starvation and EGF stimulation conditions compared to scramble control MDA-MB-231 cells (Fig. 5c), which is consistent with the interaction between CGRRF1 and EGFR in both starvation and EGF stimulation conditions (Fig. 4c). The effect of CGRRF1 knockdown on EGF-stimulated K48-linked EGFR ubiquitination was also seen in another cell line BT-549 (Additional file 5: Figure S5A). Conversely, overexpression of CGRRF1 enhanced EGFR K48-linked ubiquitination after EGF stimulation (Fig. 5d). These results indicate that CGRRF1 promotes K48 linkage-specific ubiquitination of EGFR.

In addition to K48-linked EGFR ubiquitination, we also examine the effect of CGRRF1 on K0- and K63-linked EGFR ubiquitination. In both normal growing and EGF stimulation conditions, knockdown of CGRRF1 inhibited K0-linked EGFR ubiquitination (Additional file 5: Figure S5B and S5C). This data is consistent with a role of CGRRF1 for EGFR ubiquitination. Surprisingly, we detected a smear pattern of K0-linked EGFR ubiquitination. HA-UbK0 functions as a monoubiquitin and as a ubiquitin chain terminator. The reason why we detected a smear pattern could be because that HA-UbK0 can be attached to the end of other polyubiquitination chains and/or there are multiple monoubiquitin conjugations. The CGRRF1 status did not affect K63-linked EGFR ubiquitination in normal growing condition (Additional file 5: Figure S5D and S5E). However, with EGF stimulation, overexpression of CGRRF1 enhanced K48-linked, but inhibited K63-linked, EGFR ubiquitination (Additional file 5: Figure S5F), supporting that CGRRF1 promotes K48 linkage-specific, but not K63 linkage-specific, ubiquitination of EGFR.

### RING-domain mutant CGRRF1 interacts with EGFR but fails to promote K48-linked EGFR ubiquitination

**Fig. 6:**
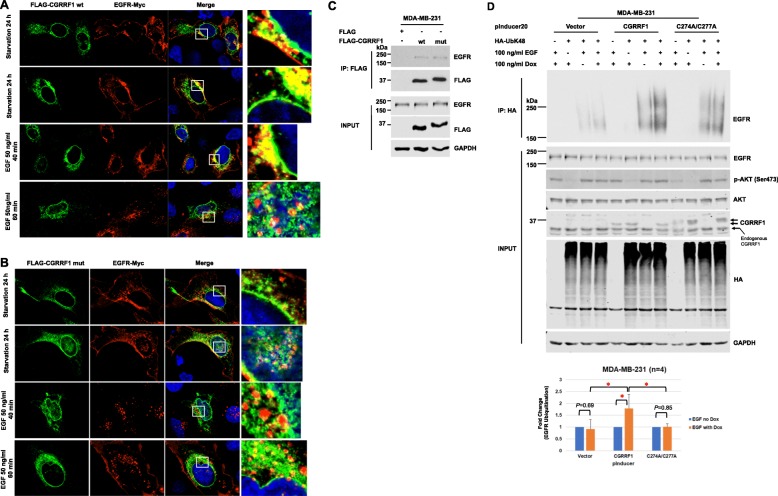
RING-domain mutant CGRRF1 (C274A/C277A) interacts with EGFR but does not ubiquitinate EGFR. **a**, **b** Immunofluorescence analysis (×100) shows colocalization of FLAG-tagged CGRRF1 (green) and Myc-tagged EGFR (red) in both starvation and EGF stimulation conditions. White blank boxes (right columns) show high magnification of CGRRF1 and EGFR colocalization from merge columns. U2OS cells were transiently co-transfected with FLAG-tagged wild-type CGRRF1 (**a**) or mutant CGRRF1 (**b**) with Myc-tagged EGFR for 24 h. Transfected cells were starved in serum-free, 0.1% BSA-containing medium for 24 h and then stimulated with EGF (50 ng/ml). **c** MDA-MB-231 cells were transfected with FLAG-tagged wild-type or mutant CGRRF1. Cells were harvested 48 h after transfection, and lysates were pulled down with anti-FLAG beads. **d** CGRRF1 doxycycline-inducible MDA-MB-231 cells were transfected with pcDNA3 or HA-UbK48. The next day after transfection, cells were serum-starved and treated with 100 ng/ml doxycycline. Twenty-four hours after starvation, cells were stimulated with 100 ng/ml EGF for 5 min. Lysates were then harvested for in vivo ubiquitination assay. Induced CGRRF1 proteins were indicated by arrows. The bar graph represents data from four independent experiments. Error bars represent mean ± SD. *p < 0.05

To elucidate whether CGRRF1-induced K48-linked EGFR ubiquitination is due to the E3 ligase activity of CGRRF1, we first examined the interaction between EGFR and RING-domain mutant CGRRF1. From immunofluorescence staining, the subcellular localization patterns between wild-type and mutant CGRRF1 were essentially indistinguishable (Fig. 6a, b). Moreover, we observed colocalization of FLAG-tagged CGRRF1 (both wild-type and mutant) and Myc-tagged EGFR in both starvation and EGF stimulation conditions (Fig. 6a, b). We also performed co-immunoprecipitation assay to verify the interaction between mutant CGRRF1 and EGFR. In HEK293T cells, mutant CGRRF1 was able to interact with GFP-tagged EGFR as proficient as wild-type CGRRF1 (Additional file 6: Figure S6). A similar result was observed in MDA-MB-231 cells in which both FLAG-tagged wild-type and mutant CGRRF1 interacted with endogenous EGFR to a similar degree (Fig. 6c), suggesting that the mutations we made on the RING domain of CGRRF1 do not affect its ability to bind to EGFR.

We then performed in vivo ubiquitination assay to investigate whether our mutant CGRRF1 fails to ubiquitinate EGFR. Upon EGF stimulation, doxycycline-induced wild-type CGRRF1 was able to enhance K48-linked EGFR ubiquitination as compared to no doxycycline control. However, doxycycline-induced mutant CGRRF1 did not promote K48-linked ubiquitination of EGFR (Fig. 6d). Since these are different stable cell lines with endogenous CGRRF1, their EGF-induced EGFR ubiquitination in the absence of doxycycline may vary depending on many factors including the levels of endogenous E3 ligases and other undefined changes acquired during cell line establishment; therefore, we compared the samples without and with doxycycline within the same cell line. In this way, the only difference between both samples is the presence or absence of the exogenous CGRRF1 that was induced by doxycycline. The data from four independent experiments are very consistent and show that only induction of wild-type CGRRF1 caused an increase in EGFR ubiquitination (Fig. 6d, bottom panel). Together, these data support that CGRRF1 is an E3 ubiquitin ligase for EGFR.

### Knockout of CGRRF1 increases EGFR protein level

**Fig. 7:**
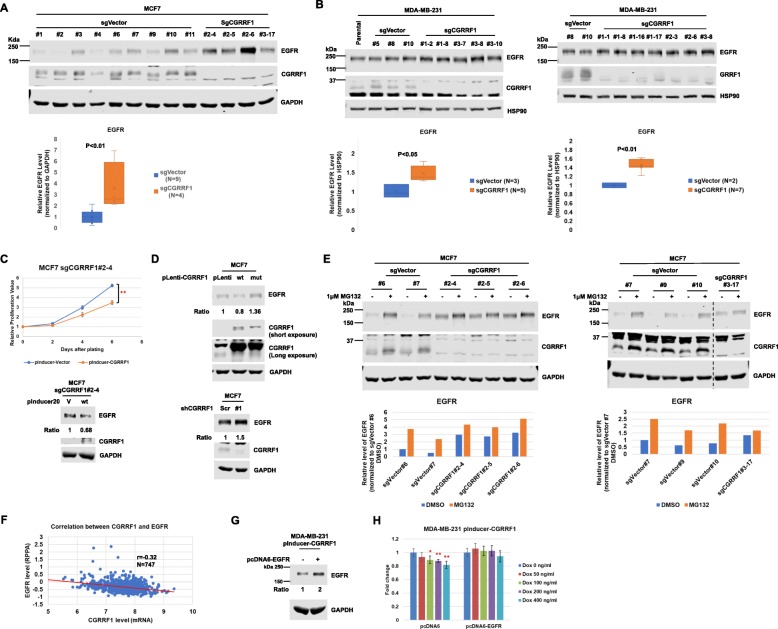
CGRRF1 regulates EGFR stability. **a**, **b** CGRRF1 was knocked out by CRISPR in MCF7 cells (**a**) and in MDA-MB-231 cells (**b**). The #number in sgVector denotes the ID of individual clones. The first number in sgCGRRF1 #number-number indicates the ID of sgCGRRF1 (e.g., #2-3 and #2-6 were generated from sgCGRRF1 oligos 2R and 2F), and the second number indicates the ID of the individual clones generated from each sgCGRRF1. EGFR expression in the CRISPR control (sgVector) and CGRRF1-knockout clones was examined by western blot analysis. **c** The expression of CGRRF1 in the CGRRF1-knockout MCF7 (sgCGRRF1#2-4) cell line was rescued by infecting with CGRRF1-expressing lentivirus. Rescue of CGRRF1 in these cells was confirmed by western blot analysis (lower panel). The growth rate of these cell lines was determined by MTT assay. Error bars represent mean ± SD (*n* = 5). **p < 0.001. **d** EGFR levels in CGRRF1-overexpressing (upper panel) and CGRRF1-knockdown (lower panel) MCF7 cell lines were examined by western blot analysis. **e** CGRRF1-knockout MCF7 cell lines were treated with 1 μM MG132 for 24 h. Lysates were harvested for western blot analysis to examine the expression of EGFR. **f** Correlation between CGRRF1 mRNA levels (RNA-seq) and EGFR protein levels (RPPA) in the TCGA breast cancer cohort. *R* = −0.32, *p* < 0.001, *n* = 747. The data in the TCGA breast cancer (BRCA) study were accessed from xenabrowser.net. **g** Establishment of EGFR overexpression by pcDNA6-EGFR in pInducer-CGRRF1 (wild-type) MDA-MB-231 cells. **h** The growth rate of pInducer-CGRRF1 (wild-type) MDA-MB-231 cells that overexpressed with and without pcDNA6-EGFR was determined by MTT assay. Cells were treated with different doses of doxycycline for 5 days. Error bars represent mean ± SD (*n* = 8). *p < 0.01, **p < 0.001

K48-linked polyubiquitination has been known for regulating proteasome-mediated protein degradation. To determine a role for CGRRF1 in the regulation of EGFR, we generated CGRRF1-knockout clones in MCF7 cells and MDA-MB-231 cells using three different sgRNAs against CGRRF1. We also generated multiple sgVector control clones for each cell line at the same time for a more robust comparison with CGRRF1-knockout clones. Indeed, CGRRF1-knockout MCF7 cells (sgCGRRF1) had higher EGFR protein levels compared to the control cell lines (sgVector) (Fig. 7a). Although not as obvious as in MCF7, knockout of CGRRF1 in MDA-MB-231 also increased EGFR expression (Fig. 7b). We then performed growth assay in these knockout cells. Our results showed that knockout of CGRRF1 increased cell proliferation (Additional file 7: Figure S7A and S7B), which is consistent with Fig. 1. We also rescued CGRRF1 expression in one of the CGRRF1-knockout MCF7 cell lines and then performed proliferation assay. As shown in Fig. 7c, CGRRF1-rescued cells grew slower than CGRRF1-knockout cells. Besides, we noticed that the EGFR expression is decreased in CGRRF1-rescued cells (Fig. 7c, bottom panel). A similar result was observed in wild-type, but not mutant, CGRRF1-overexpressing MCF7 cell lines (Fig. 7d). We also checked the EGFR level in CGRRF1-knockdown cells. Just like the knockout cell lines, knockdown of CGRRF1 increased EGFR expression (Fig. 7d, bottom panel).

To further understand whether the increased EGFR in CGRRF1-knockout cell lines is due to stabilization of EGFR protein, we treated CGRRF1-knockout cells with a proteasome inhibitor, MG132, and compared the expression of EGFR. As shown in Fig. 7e, control cell lines had more induction of EGFR after MG132 treatment than CGRRF1-knockout cell lines. These data support that lower EGFR expression in control cell lines is due to CGRRF1-mediated EGFR proteasomal degradation. In the TCGA database, we also found that there is a negative correlation between CGRRF1 mRNA and EGFR protein levels (Fig. 7f). Although the sample number is small, there is a very significant negative correlation (*r* = −0.56) between CGRRF1 and EGFR protein levels in the breast cancer cell lines (Additional file 8: Figure S8). This further supports the idea that CGRRF1 regulates EGFR expression.

To examine whether CGRRF1 inhibits cell growth at least in part through EGFR, we established EGFR-overexpressing cell lines in wild-type CGRRF1 doxycycline-inducible MDA-MB-231 cells (Fig. 7g). As shown in Fig. 7h, the control cells (pcDNA6) grew slower after inducing the expression of CGRRF1 by doxycycline. However, the growth rate of the cells overexpressing EGFR was not affected by the induction of CGRRF1. This data suggests that CGRRF1 inhibits cell growth at least in part by decreasing the expression of EGFR.

### CGRRF1 regulates AKT phosphorylation and EGFR nuclear translocation

**Fig. 8:**
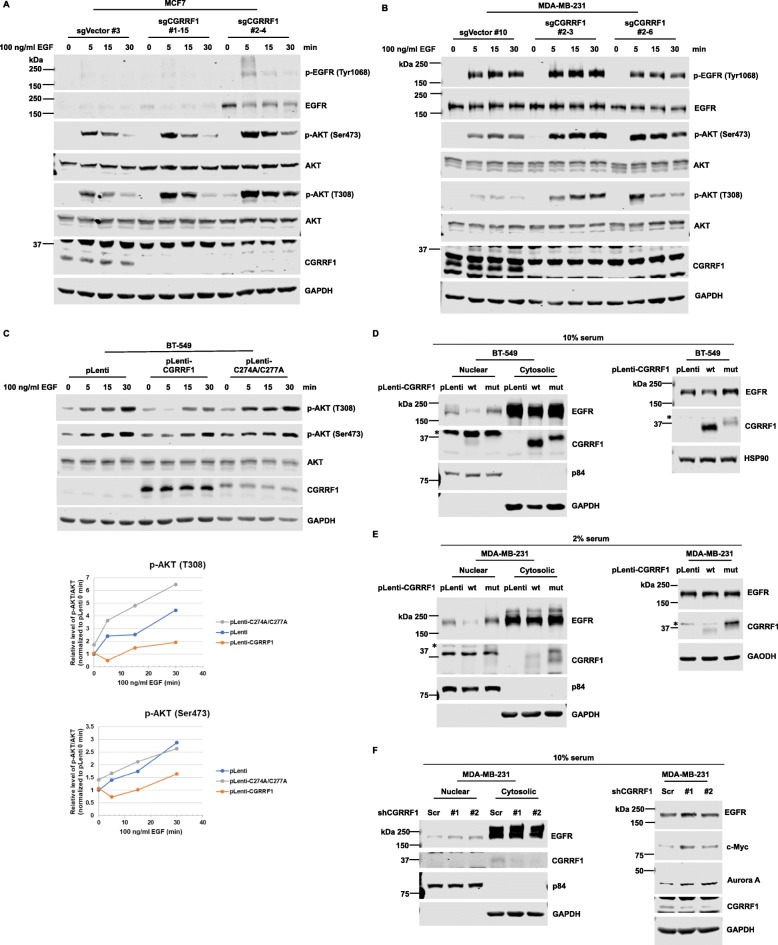
CGRRF1 regulates AKT phosphorylation and the expression of nuclear EGFR. **a**, **b** AKT phosphorylation after EGF stimulation in CGRRF1-knockout MCF7 cells (**a**) and MDA-MB-231 cells (**b**) was examined by western blot analysis. **c** EGF-induced AKT phosphorylation in CGRRF1-overexpressing BT-549 cells was examined by western blot analysis. The quantification of AKT phosphorylation is shown in the bottom graphs. **d**–**f** Subcellular fractionation experiments were performed to examine the levels of nuclear EGFR in CGRRF1-overexpressing BT-549 cells (**d**), CGRRF1-overexpressing MDA-MB-231 cells (**e**), and CGRRF1-knockdown MDA-MB-231 cells (**f**). The experiment in **e** was carried out under 2% serum condition to increase the expression of mutant CGRRF1. p84 was used as a nuclear marker, and GAPDH was used as a cytosolic marker. We also harvested parallel plates for total protein lysates (right panels of **d**–**f**). Nonspecific bands are marked with asterisks (*)

EGFR contains a tyrosine kinase domain. Upon ligand stimulation, EGFR can form homo- or hetero-dimer and auto-phosphorylate itself. This will lead to the activation of RAS/MAPK, PI3K/AKT, PLC-PKC, and Jak/STAT pathways. To determine whether CGRRF1 regulates EGFR signaling, we checked the downstream signaling pathways of EGFR. Indeed, knockout of CGRRF1 increased AKT phosphorylation after EGF stimulation in both MCF7 cells (Fig. 8a) and MDA-MB-231 cells (Fig. 8b). Interestingly, while the enhanced AKT phosphorylation in CGRRF1-knockout MCF7 cells is at least in part due to higher EGFR protein levels in these cells, the EGFR levels were not significantly changed in CGRRF1-knockout MDA-MB-231 cells under this experiment. We also examine AKT phosphorylation in CGRRF1-overexpressing BT-549 cell lines. Overexpression of wild-type but not mutant CGRRF1 inhibited AKT phosphorylation (Fig. 8c). These data suggest that the ubiquitination of EGFR by CGRRF1 might also regulate EGFR signaling function besides its protein level.

EGFR also contains a nuclear localization sequence (NLS), which plays an important role in nuclear translocation [9]. Cell surface EGFR is translocated to the nucleus through COPI-mediated retrograde transport from the Golgi to the ER, then shuttled from the outer nuclear membrane (ONM) to the inner nuclear membrane (INM), and finally released from the INM to the nucleoplasm [10]. Since CGRRF1 has been reported to localize to the ER, CGRRF1 might be involved in EGFR nuclear translocation. To address this possibility, we made use of the TNBC cell lines BT-549 and MDA-MB-231, in which the total EGFR levels were only modestly affected by CGRRF1 expression or knockdown (Fig. 8d, right panel, and Fig. 8f, right panel). Indeed, the fraction of nuclear EGFR was significantly lower in wild-type CGRRF1-overexpressing BT-549 cells than in the control and mutant CGRRF1-overexpressing cells (Fig. 8d). In this experiment, cells were cultured in medium containing 10% serum; therefore, the expression level of mutant CGRRF1 was less than that of the wild type. To overcome this problem and to exclude the possibility that the lack of effect by mutant CGRRF1 was due to its lower expression, we cultured CGRRF1-overexpressing MDA-MB-231 cells in low serum condition (2% serum) for a few days to increase the expression of mutant CGRRF1, as we observed in Additional file 2: Figure S2B. Indeed, the nuclear EGFR was significantly decreased in wild-type but not in mutant CGRRF1-overexpressing MDA-MB-231 cell lines, despite mutant CGRRF1 being expressed at a higher level than the wild type (Fig. 8e). On the other hand, we detected higher nuclear EGFR fraction in CGRRF1-knockdown MDA-MB-231 cells (Fig. 8f). Correspondingly, the targets of nuclear EGFR, c-Myc [11] and Aurora A [12], were expressed at higher levels in CGRRF1-knockdown MDA-MB-231 cells (Fig. 8f, right panel). These results suggest that in addition to regulating EGFR stability, CGRRF1 might regulate the nuclear translocation of EGFR through its ubiquitin E3 ligase activity.

### Low CGRRF1 expression in breast cancer is associated with poor patient survival

**Fig. 9:**
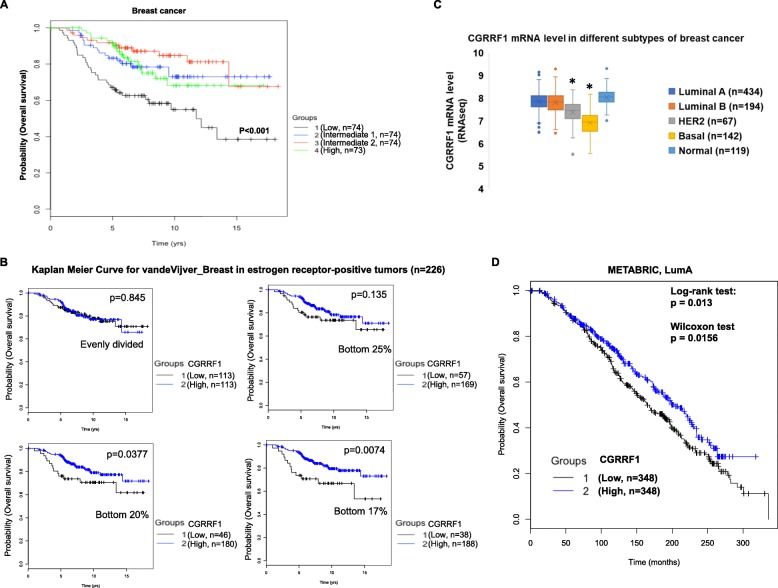
Breast cancer patients with lower CGRRF1 have poor overall survival. **a** Kaplan-Meier analysis of breast cancer according to CGRRF1 expression levels. The CGRRF1 expression levels and clinical outcomes of a published dataset [13] were extracted from Oncomine. Patients were ranked according to the CGRRF1 expression levels in their breast tumors. We then divided the patients into four groups (CGRRF1 levels: high > intermediate 2 > intermediate 1 > low). The numbers (*n*) of patients in each group are indicated. *n* was assigned so that each group had almost an equal number of patients. Kaplan-Meier curves were then derived from each group. The Wilcoxon test was used to evaluate significance. **b** Estrogen receptor-positive breast cancer patients in the van de Vijver dataset [13] were analyzed. Patients were ranked according to CGRRF1 mRNA levels and divided into two groups according to the indicated methods for the Kaplan-Meier curve analysis: (1) equally divided; (2) low CGRRF1 expression (bottom 1/4 of the total cohort) vs. the remaining 3/4 of the cohort; (3) low CGRRF1 expression (bottom 1/5 of the total cohort) vs. the remaining 4/5 of the cohort; (4) low CGRRF1 expression (bottom 1/6 of the total cohort) vs. the remaining 5/6 of the cohort. The Wilcoxon test was used to evaluate significance. *P* values for the tests between the groups are indicated. **c** *CGRRF1* transcript levels in different subtypes of breast cancer (data extracted from UCSC Xena, TCGA breast cancer (BRCA)). The asterisk (*) means *p* < 0.001 as compared to other subtypes. **d** Kaplan-Meier curves in Luminal A breast cancer patients from the METABRIC cohort. Patients were equally separated into two groups based on the expression of CGRRF1 in their breast cancers. *P* values from both the log-rank test and Wilcoxon test are shown

Given the growth suppressor role of CGRRF1 in breast cancer cell lines and the xenograft model shown above, we investigated publicly available breast cancer datasets to correlate CGRRF1 expression with patient survival. Kaplan-Meier survival analysis from the van de Vijver database (stages I and II breast cancer) [13] shows that breast cancer patients with low CGRRF1 expression in their breast tumors had poor survival (Fig. 9a). CGRRF1 expression is lower in estrogen receptor-negative breast cancers than in estrogen receptor-positive cancers (Oncomine). To avoid this confounding factor, we analyzed only estrogen receptor-positive breast cancers in the van de Vijver cohort. As shown in Fig. 8b, the lower the CGRRF1 levels are, the shorter the patient survived. The number of estrogen receptor-negative patients (*n* = 69) in the van de Vijver cohort is too small to perform analysis. We also found similar results in patients with kidney carcinoma or lung adenocarcinoma (Additional file 9: Figure S9A), suggesting a general role for CGRRF1 in suppressing tumor growth.

There are five major subtypes of breast cancer. We also checked the expression of CGRRF1 in each subtype. In the TCGA breast cancer cohort, both HER2-positive and basal-like breast cancer patients had significantly lower expression of CGRRF1 compared to other subtypes (Fig. 9c). Basal-like breast cancer patients have the worst survival, so the association of low CGRRF1 with poor survival could be in part due to the fact that some cancers with low CGRRF1 expression are basal-like subtype. To further investigate whether CGRRF1 expression bears prognostic significance within the same subtype of breast cancer, we used the KM Plotter server to perform analysis of each subtype from a large number of datasets. We found that lower expression of CGRRF1 is also associated with a shorter patient overall survival in the Luminal A subtype and HER2-positive subtype of breast cancer (Additional file 9: Figure S9B). We do not see this association in the basal-like cohort, probably because most basal-like breast cancers already express low levels of CGRRF1. The association between low CGRRF1 expression and shorter patient survival is also seen in the Luminal A subtype in the METRBRIC dataset (Fig. 8d). Thus, analyses from multiple datasets show an association between low CGRRF1 expression and poor patient survival.

### The expression of CGRRF1 is often downregulated in breast carcinoma due to promoter hypermethylation

**Fig. 10:**
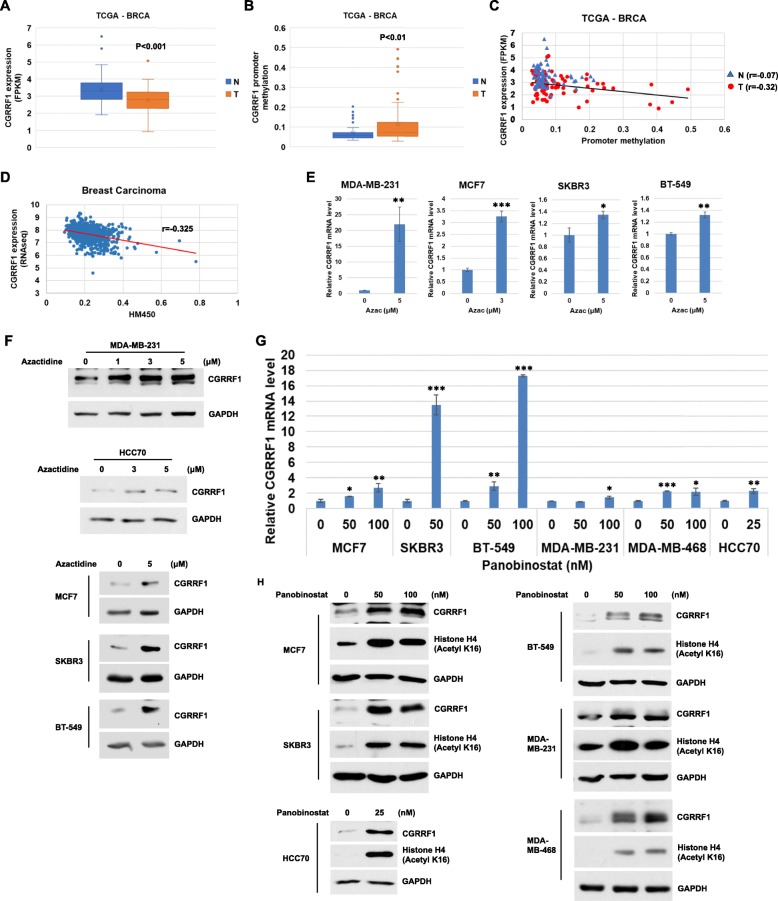
Epigenetic modification regulates the expression of CGRRF1. **a** CGRRF1 is significantly decreased at the transcript levels in breast tumor samples (T) as compared to matched normal breast tissues (N) (data extracted from TCGAportal, TCGA BRCA, *n* = 76 pairs). **b** *CGRRF1* promoter methylation increased in tumor samples (T) as compared to matched normal breast tissue (N) (data extracted from TCGAportal, TCGA BRCA, *n* = 76 pairs). **c** An inverse correlation between CGRRF1 mRNA levels and *CGRRF1* promoter methylation (chr14:54509516) in breast tumor tissues (T), but not in normal breast (N) (data extracted from TCGAportal, TCGA BRCA, *n* = 76 pairs). The black line (regression line) represents the correlation of tumor tissues. “*r*” represents the Pearson correlation coefficient. **d** An inverse correlation between CGRRF1 mRNA levels and *CGRRF1* promoter methylation (HM450, cg18492804) in TCGA breast carcinoma cohort (data extracted from UCSC Xena, TCGA breast cancer (BRCA), *n* = 888). **e**, **f** Treatment of 5-azacitidine (72 h) induced CGRRF1 mRNA (**e**) and protein (**f**) expression in a panel of breast cancer cell lines. **g**, **h** Panobinostat treatment for 24 h induced *CGRRF1* transcripts (**g**) and proteins (**h**) in a panel of breast cancer cell lines. The expression of CGRRF1 mRNA was measured by qRT-PCR, and the protein level was measured by western blot. Histone H4 K16 acetylation was measured to serve as a positive control for panobinostat treatment. *p < 0.05, **p < 0.01, ***p < 0.001

Comparing the transcript levels of CGRRF1 between matched normal breast tissues and breast tumors in the TCGA breast cancer cohort, we noticed that CGRRF1 expression is significantly lower in tumor tissues (Fig. 10a and Additional file 10: Figure S10A). Aberrant epigenetic modifications, such as changes in DNA methylation and histone modification, are associated with the development and progression of cancer. To understand whether the decrease in CGRRF1 in breast carcinoma is caused by alterations in epigenetic modifications, we examined the methylation status of the promoter of CGRRF1 in the TCGA breast cancer cohort. Indeed, there is a significant increase in *CGRRF1* promoter methylation in breast tumors compared to that in normal breast tissues (Fig. 10b and Additional file 10: Figure S10B). We further analyzed the correlation between *CGRRF1* transcript levels and its promoter methylation status in the TCGA breast cancer cohort. In normal breast tissues, there is no correlation between the expression of CGRRF1 and its promoter methylation (*r* = −0.07). However, there is a negative correlation in the matched 76 breast tumor samples (*r* = −0.32) (Fig. 10c) and in 888 TCGA breast cancer tissues (*r* = −0.325) (Fig. 10d). In addition, similar negative correlation between CGRRF1 expression and promoter methylation is found in other types of cancer (Additional file 10: Figure S10C). These data suggested the possibility that changes in *CGRRF1* promoter methylation regulate the expression of CGRRF1 and are involved in the development of breast cancer.

To test the epigenetic mechanism for downregulation of CGRRF1 in breast cancer, we treated a panel of breast cancer cell lines with a demethylation agent, 5-azactidine. Indeed, 5-azacitidine treatment increased the *CGRRF1* transcripts (Fig. 10e) and protein expression (Fig. 10f) in breast cancer cells. We also treated these cells with a histone deacetylase (HDAC) inhibitor, panobinostat. CGRRF1 expression was induced at both the mRNA level (Fig. 10g) and protein level (Fig. 10h) after the treatment. On the other hand, EGFR expression was decreased after the treatment (Additional file 11: Figure S11). Together, these results indicated that epigenetic alterations in the *CGRRF1* promoter lead to its downregulation in breast cancer.

## Discussion

In this study, we demonstrate the in vitro and in vivo growth-inhibitory activity of an ER-resident E3 ubiquitin ligase, CGRRF1, in breast cancer and identify EGFR as its ubiquitin ligase substrate through unbiased RPPA analysis. The ubiquitination of EGFR promoted by CGRRF1 leads to EGFR degradation and inhibition of its signaling function and nuclear translocation. Since EGFR is a driver of tumorigenesis [14], hyperactivation of EGFR signaling pathways caused by *CGRRF1* promoter hypermethylation and downregulation seen in many types of cancer may contribute to cancer progression.

Previous studies demonstrated that CGRRF1 ubiquitinates and regulates the stability of Evi, a Wnt cargo receptor regulating Wnt protein secretion [4, 15]. Our study identifies EGFR as a new substrate of CGRRF1. Although CGRRF1 could potentially regulate other proteins/receptors, overexpression of EGFR can mitigate the growth-inhibitory effect of CGRRF1, supporting EGFR regulation as an important mechanism for the suppressor function of CGRRF1.

EGFR can be ubiquitinated by several E3 ligases. c-cbl has been reported as a RING-finger E3 ubiquitin ligase of receptor tyrosine kinases, including EGFR, platelet-derived growth factor receptor (PDGFR) [16], fibroblast growth factor receptor (FGFR) [17], and vascular endothelial growth factor receptor (VEGFR) [18]. c-cbl is responsible for ligand-induced ubiquitination that is important for receptor internalization and endocytosis, leading to lysosomal degradation [19, 20]. c-cbl-induced EGFR ubiquitination is mainly through K63 linkage [19, 21]. EGFR can also be ubiquitinated by RNF144A in a ligand-dependent manner to regulate EGFR vesicular transport and sustain EGFR signaling [7]. Unlike c-cbl-regulated ligand-induced EGFR K63 linkage ubiquitination, CGRRF1 ubiquitinates EGFR by K48 linkage. Interestingly, the EGF-induced K63 linkage EGFR ubiquitination is reduced upon CGRRF1 overexpression, raising possibilities of cross-regulation between different linkages of ubiquitination. Contrary to c-cbl and RNF144A, the interaction between CGRRF1 and EGFR does not require ligand stimulation. Prior studies have demonstrated that CGRRF1 localizes on the ER membrane [5, 6]; thus, CGRRF1 might regulate the stability of EGFR through ERAD. The ER is responsible for protein synthesis; EGFR synthesized in the ER with appropriate conformation can proceed to the Golgi apparatus and then to the plasma membrane to regulate EGFR signaling. Since CGRRF1 regulates EGFR stability in the ER, it is possible that CGRRF1 affects the expression of cell surface EGFR and its downstream signaling. Indeed, there was a higher level of AKT phosphorylation after EGF stimulation in CGRRF1-knockout cells.

Recently, studies have shown that EGFR can be translocated to the nucleus. EGFR nuclear translocation is regulated by a retrograde trafficking from the Golgi to the ER [10]. We detected a lower level of nuclear EGFR when cells overexpressed wild-type CGRRF1. This suggests that in addition to regulating the expression of EGFR in the plasma membrane, CGRRF1 might also regulate the expression or translocation of nuclear EGFR. Interestingly, we detected the expression of CGRRF1 in the nuclear fraction. Since CGRRF1 contains a transmembrane domain, CGRRF1 might localize on the nuclear membrane. Indeed, we also observed nuclear membrane localization of CGRRF1 and colocalization between CGRRF1 and EGFR in the ER close to the nuclear membrane (Fig. 6a, b). The immunofluorescence data are consistent with the regulation of EGFR nuclear transport by CGRRF1. The function of EGFR in the nucleus is distinct from that in the plasma membrane. Nuclear EGFR can function as a co-transcription factor and regulates the expression of c-fos, cyclin D1, inducible nitric oxide synthase (iNOS), B-Myb, cyclooxygenase-2 (COX-2), and Aurora A [12, 22–25]. Nuclear EGFR also phosphorylate proliferating cell nuclear antigen (PCNA) to enhance cell proliferation [26]. Upon radiation treatment, nuclear EGFR interacts with and activates DNA-dependent protein kinase (DNAPK). Activation of DNAPK results in increase in c-Myc mRNA, which contributes to cell survival and radioresistance, by suppressing the activity of PNPase [27]. Since our results showed that CGRRF1 regulates the levels of nuclear EGFR, CGRRF1-regulated growth suppression might be through these mechanisms. Indeed, knockdown of CGRRF1 increased the expression of Aurora A and c-Myc (Fig. 8f), which is consistent with higher expression of nuclear EGFR in knockdown cells.

In addition to EGFR, our RPPA analysis identified other 12 proteins which were regulated by CGRRF1 in MDA-MB-231 xenografts. Among them, pS15-p53 was induced by both wild-type and mutant CGRRF1. On the contrary, pS1981-ATM was repressed by both wild-type and mutant CGRRF1. P-ERK1/2 was also induced by both wild-type and mutant CGRRF1. Although not statistically significant, p-p38(T180/Y182) was induced by wild-type CGRRF1. Thus, it is possible that expression of CGRRF1 protein caused some yet-to-be-defined stress(es) (not DNA damage) in MDA-MB-231 xenografts. Furthermore, whether CGRRF1 regulates the other two receptors, PDGFRβ and Axl, also deserves future investigation.

While the growth-suppressive activity of CGRRF1 is largely dependent on its E3 ligase function, the RING-domain mutant CGRRF1 did not totally lose the growth-inhibitory activity in many experiments when it was stably and constitutively expressed. While it is formally possible that the C274A/C277A mutant CGRRF1 still exhibits a low level of residual E3 ligase activity, we did not observe any enhancement of EGFR ubiquitination when inducing the expression of this mutant (Fig. 6d). RING-domain mutant CGRRF1 was less stable than wild-type CGRRF1. Thus, the mutant might be structurally unstable, and constitutive overexpression of this mutant protein might indirectly confer some growth disadvantage compared with the empty vector control during the establishment of the stable cell line. This possibility is supported by the finding that mutant CGRRF1 indeed did not inhibit growth when its expression was induced by doxycycline, suggesting that the effect seen in the constitutive expression system may not be a direct effect. However, we cannot rule out the possibility that CGRRF1 might have other E3 ligase-independent activity as some E3 ligases, e.g., EDD/UBR5 [28]. It is worth pointing out that doxycycline-induced wild-type CGRRF1 did not cause apparent growth arrest in the first few days, indicating that CGRRF1-mediated growth suppression takes time to manifest its effects. These data suggest that the mechanism of CGRRF1-mediated growth suppression is unlikely similar to other cell cycle checkpoint proteins, but rather through regulation of ERAD of growth receptors, etc.

Previous studies demonstrated that CGRRF1 is a target gene of p53 and RBL2/p130 [1, 3]. In addition to transcriptional regulation, expression of CGRRF1 might be affected by epigenetic modifications, which include DNA methylation, histone modification, and nucleosomal remodeling. Epigenetic regulation is important to control normal growth and development, and epigenetic aberrations can lead to cancer, neurological diseases, and autoimmune disorders [29]. In cancer, global hypomethylation has been reported to activate the expression of oncogenes [30]. On the other hand, site-specific hypermethylation of tumor suppressor genes silences gene expression [31, 32]. The expression of CGRRF1 is lower in cancer tissues, most likely due to hypermethylation of the *CGRRF1* promoter. Supporting this, CGRRF1 expression in these breast cancer cells can be enhanced by treatment with a demethylating agent, 5-azactidine, and an HDAC inhibitor, panobinostat. Histone modification regulates gene expression by altering the chromatin structure. Histone acetylation involves two enzymes, histone acetyl transferases (HATs) and histone deacetylase (HDAC). Histone acetylation induced by HATs causes euchromatin, which allows gene transcription; on the other hand, histone hypoacetylation induced by HDAC results in a more compact chromatin structure, which silences gene expression. Since epigenetic change is a reversible process and breast cancer patients with higher CGRRF1 had better survival, increasing the expression of CGRRF1 by altering the epigenetic modification might be a potential therapy that deserves future investigation.

## Conclusions

We demonstrate that CGRRF1 suppresses growth of breast cancer and its E3 ligase activity is involved in CGRRF1-mediated growth suppression. In addition to Evi, which is involved in Wnt/β-catenin signaling, we identified EGFR as a novel substrate of CGRRF1. CGRRF1-mediated EGFR ubiquitination affects EGFR stability, which might affect the expression of plasma membrane-bound EGFR and nuclear EGFR. The expression of CGRRF1 is downregulated in breast carcinoma, and breast cancer patients with lower CGRRF1 had poor survival. By analyzing the methylation status of the *CGRRF1* promoter, we show that the *CGRRF1* promoter region is hypermethylated in breast cancer tissues and demonstrate that the expression of CGRRF1 can be restored by 5-azacitidine and panobinostat, which might be considered as part of the therapy for these patients.

## Supplementary information

**Additional file 1: Figure S1.** The complete set of colony formation assay in Fig. 1E (A) and Fig. 2E (B).

**Additional file 2: Figure S2.** (A) CGRRF1-overexpressing MCF7 cell lines were treated with 100 μM cycloheximide (CHX), and then harvested at the indicated time points. The expression of CGRRF1 in the cell lysates were determined by western blot. (B) CGRRF1-overexpressing MDA-MB-231 cell lines were cultured in different percentage of fetal bovine serum. The expression of wild-type and mutant (C274A/C277A) CGRRF1 was determined by western blot.

**Additional file 3: Figure S3.** (A) The heatmap summarizes the RPPA profiling of the proteins which have significant (*p* < 0.05) difference among control, wild-type, and mutant CGRRF1 groups. (B) Ki67 level from RPPA analysis in each sample was normalized to the average of the pLenti group. Although the difference is not statistically significant, there is a trend of lower levels of Ki67 in wild-type CGRRF1 group compared with the other groups. (C) EGFR levels in xenograft lysates were examined by western blot analysis. Vinculin was used as a loading control. Nonspecific band is marked with an asterisk (*).

**Additional file 4: Figure S4.** GFP-tagged EGFR interacts with endogenous CGRRF1. GFP or GFP-EGFR was transfected into HEK293T. 48 h after transfection, lysates were prepared and pulled down with anti-GFP beads. The bound CGRRF1 was detected by western blot using anti-CGRRF1 antibody. CGRRF1 signal was indicated by an arrow.

**Additional file 5: Figure S5.** (A) CGRRF1-knockdown BT-549 cell lines were transfected with pcDNA3 or HA-UbK48. Next day, cells were serum-starved for 24 h, and then treated with 100 ng/ml EGF for 15 min. Cell lysates were harvested and in vivo ubiquitination assay was performed. (B) CGRRF1-knockdown BT-549 cell lines were transfected with pcDNA3, HA-UbK48, or HA-UbK0. Lysates were harvested 48 h after transfection and subjected to in vivo ubiquitination assay. CGRRF1 signal was indicated by an arrow, and a nonspecific band was marked with an asterisk (*). (C) CGRRF1-knockdown MDA-MB-231 cell lines were transfected with pcDNA3 or HA-UbK0. Next day, cells were serum-starved for 24 h, and then treated with 100 ng/ml EGF for 5 min. Cell lysates were harvested and in vivo ubiquitination assay was performed. (D) CGRRF1-knockdown BT-549 cell lines were transfected with pcDNA3 or HA-UbK63. Lysates were harvested 48 h after transfection and subjected to in vivo ubiquitination assay. (E) CGRRF1-overexpressing MDA-MB-231 cell lines were transfected with pcDNA3 or HA-UbK63. Lysates were harvested 48 h after transfection and subjected to in vivo ubiquitination assay. (F) T47D cells were co-transfected with FLAG-CGRRF1, HA-tagged UbK48 or UbK63. Next day, cells were serum-starved for 24 h, and then treated with 100 ng/ml EGF for 30 min. Lysates were harvested and followed by in vivo ubiquitination assay.

**Additional file 6: Figure S6.** HEK293T cells were co-transfected with GFP-EGFR, Myc-tagged wild-type or mutant CGRRF1. Lysates were harvested 48 h after transfection, and GFP-EGFR was pulled down with GFP beads, followed by immunoblotting using indicated antibodies.

**Additional file 7: Figure S7.** Knockout of CGRRF1 enhances cell proliferation. (A) The growth rate of CGRRF1-knockout MCF7 cell line (sgCGRRF1#2–5) was determined by MTT assay. Error bars represent mean ± SD (*n* = 6). *p < 0.01. (B) The growth rate of CGRRF1-knockout MDA-MB-231 cell lines were determined by MTT assay. Error bars represent mean ± SD (n = 6). *p < 0.01, **p < 0.001.

**Additional file 8: Figure S8.** Correlation between CGRRF1 and EGFR protein levels in breast cancer cell lines. The membrane in Fig. 1A was probed with EGFR and the levels between CGRRF1 and EGFR were quantified by Infrared Imaging, normalized to that in MCF7 cells and then correlated. Pearson correlation coefficient R = -0.56. For easy readability and comparison, the western blots in Fig. 1A are shown again side-by-side with EGFR blots.

**Additional file 9: Figure S9.** Cancer patients with lower CGRRF1 had poor survival. (A) Kaplan-Meier curves of the overall survival of patients with kidney renal clear cell carcinoma, kidney renal papillary cell carcinoma, or lung adenocarcinoma. Patients were separated into two groups based on the expression of CGRRF1 in their tumors (data generated using KM Plotter server, kmplot.com, with auto select best cutoff and including all datasets in the server). (B) Kaplan-Meier curves in Luminal A and HER2-positive breast cancer patients. Patients were separated into two groups based on the expression of CGRRF1 (data generated using KM Plotter (auto select best cutoff, overall survival, and including all datasets in the server)).

**Additional file 10: Figure S10.** There is a negative correlation between the CGRRF1 mRNA expression and its promoter methylation status among different cancers. (A) CGRRF1 expression in normal breast tissues (N) and matched breast tumor samples (T) (data extracted from TCGAportal, TCGA BRCA, *n* = 76 pairs). (B) CGRRF1 promoter methylation status in patients with decreased CGRRF1 expression in tumor samples (data extracted from TCGAportal, TCGA BRCA, *n* = 57 pairs). (C) Pearson correlation coefficients between CGRRF1 mRNA levels and CGRRF1 promoter methylation (HM450) in different types of cancer (data extracted from cBioPortal, TCGA provisional).

**Additional file 11: Figure S11**. Panobinostat treatment increases the protein expression of CGRRF1 but reduces EGFR protein level. Cells were harvested 24 h after the treatment of panobinostat. The expression of CGRRF1 and EGFR was measured by western blot. Histone H4 K16 acetylation served as a positive control for panobinostat treatment.

## Data Availability

All data generated for this study are included within this article and its additional files.

## Abbreviations

CGRRF1: Cell growth regulator with RING-finger domain 1
ER: Endoplasmic reticulum
ERAD: Endoplasmic reticulum-associated degradation
PDI: Protein disulfide isomerase
Evi (Wls/CPR177): Evenness interrupted homolog (Wntless Wnt ligand secretion mediator/G protein-coupled receptor 177)
RPPA: Reverse phase protein array
RT-qPCR: Reverse transcription quantitative polymerase chain reaction
EGF: Epidermal growth factor
EGFR: Epidermal growth factor receptor
HER2: Human epidermal growth factor receptor 2
TNBC: Triple-negative breast cancer
RBL2: Retinoblastoma-like protein 2
HDAC: Histone deacetylase
E2: Ubiquitin-conjugating enzyme
FPKM: Fragments per kilobase million
METABRIC: Molecular Taxonomy of Breast Cancer International Consortium
