# Supplementary figures and images for: CGRRF1, a growth suppressor, regulates EGFR ubiquitination in breast cancer
Source: Breast Cancer Res. 2019 Dec 4;21:134. doi: 10.1186/s13058-019-1212-2 (PMC6894136; doi:10.1186/s13058-019-1212-2)

**A**

**BT-549**

shScr

shCGRRF1#1

shCGRRF1#2

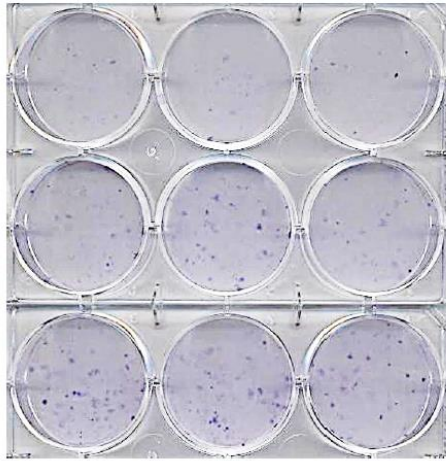

**B**

**SKBR3**

**Supplementary Figure 1**

pLenti

pLenti-  
CGRRF1

pLenti-  
C274A/C277A

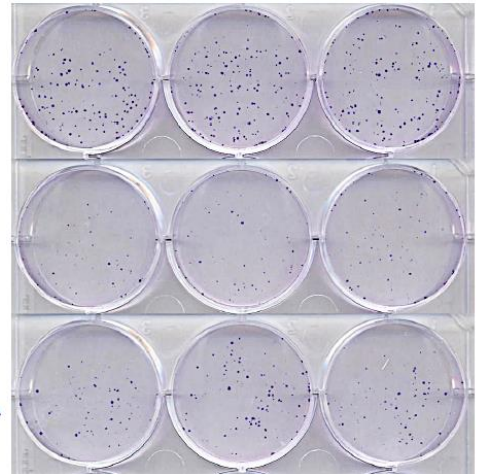

Supplement: Supplementary file 1 — Additional file 1: Figure S1. The complete set of colony formation assay in Fig. 1E (A) and Fig. 2E (B). [file 13058_2019_1212_MOESM1_ESM.pdf]

Supplementary Figure 2

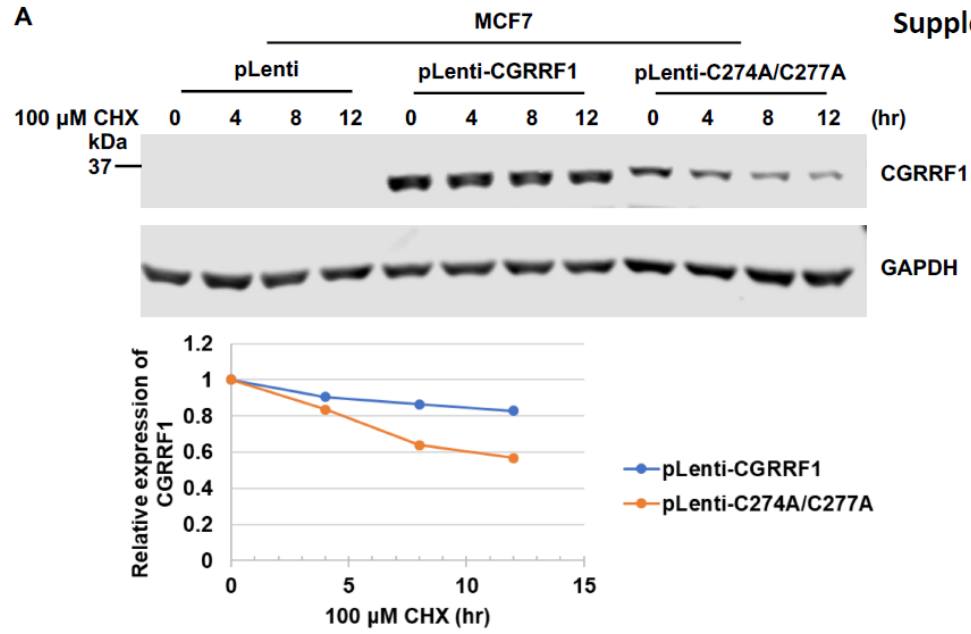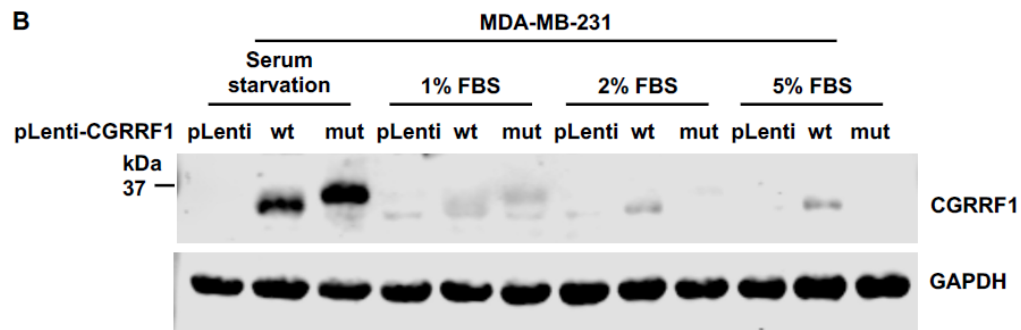

Supplement: Supplementary file 2 — Additional file 2: Figure S2. (A) CGRRF1-overexpressing MCF7 cell lines were treated with 100 μM cycloheximide (CHX), and then harvested at the indicated time points. The expression of CGRRF1 in the cell lysates were determined by western blot. (B) CGRRF1-overexpressing MDA-MB-231 cell lines were cultured in different percentage of fetal bovine serum. The expression of wild-type and mutant (C274A/C277A) CGRRF1 was determined by western blot. [file 13058_2019_1212_MOESM2_ESM.pdf]

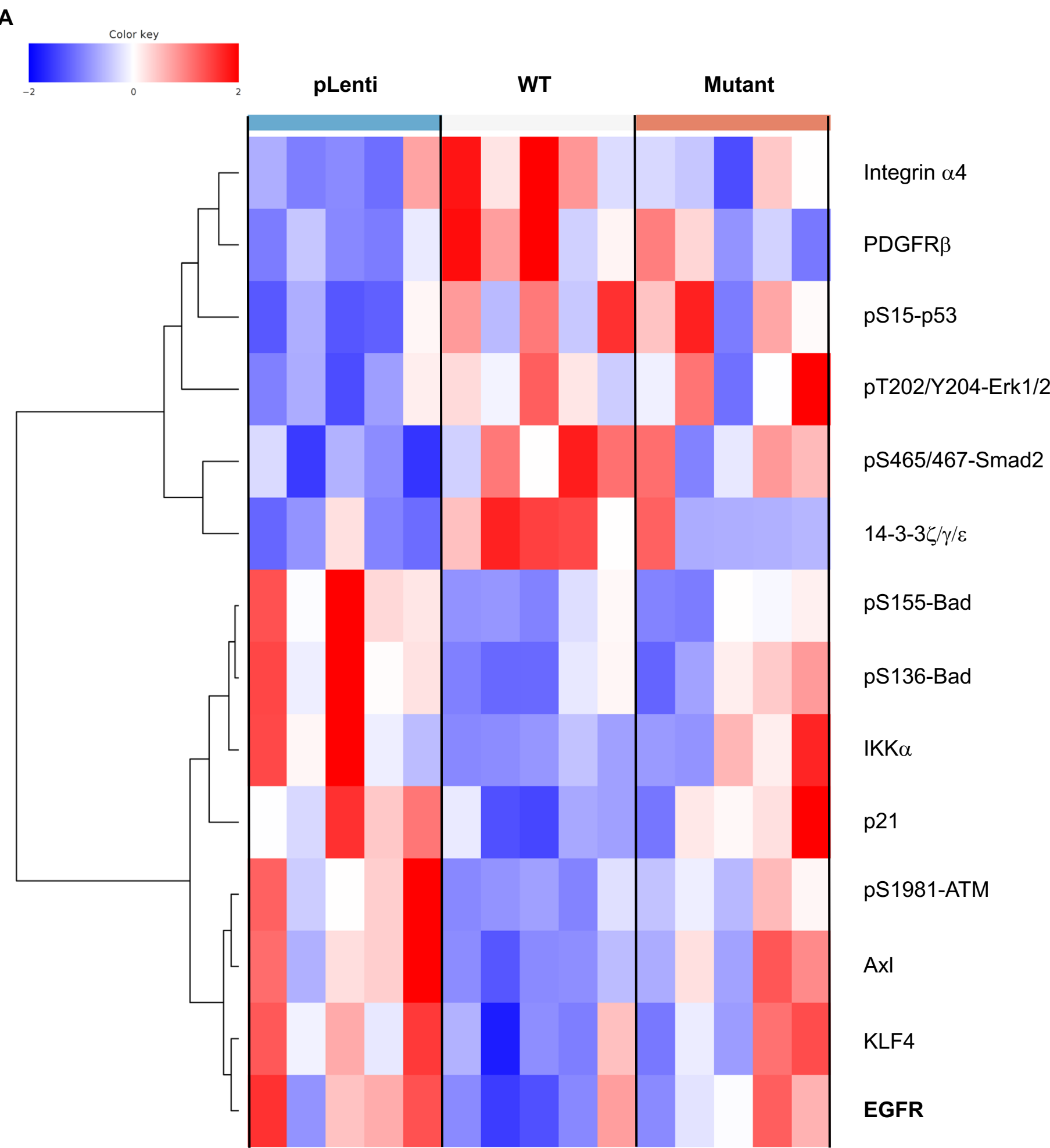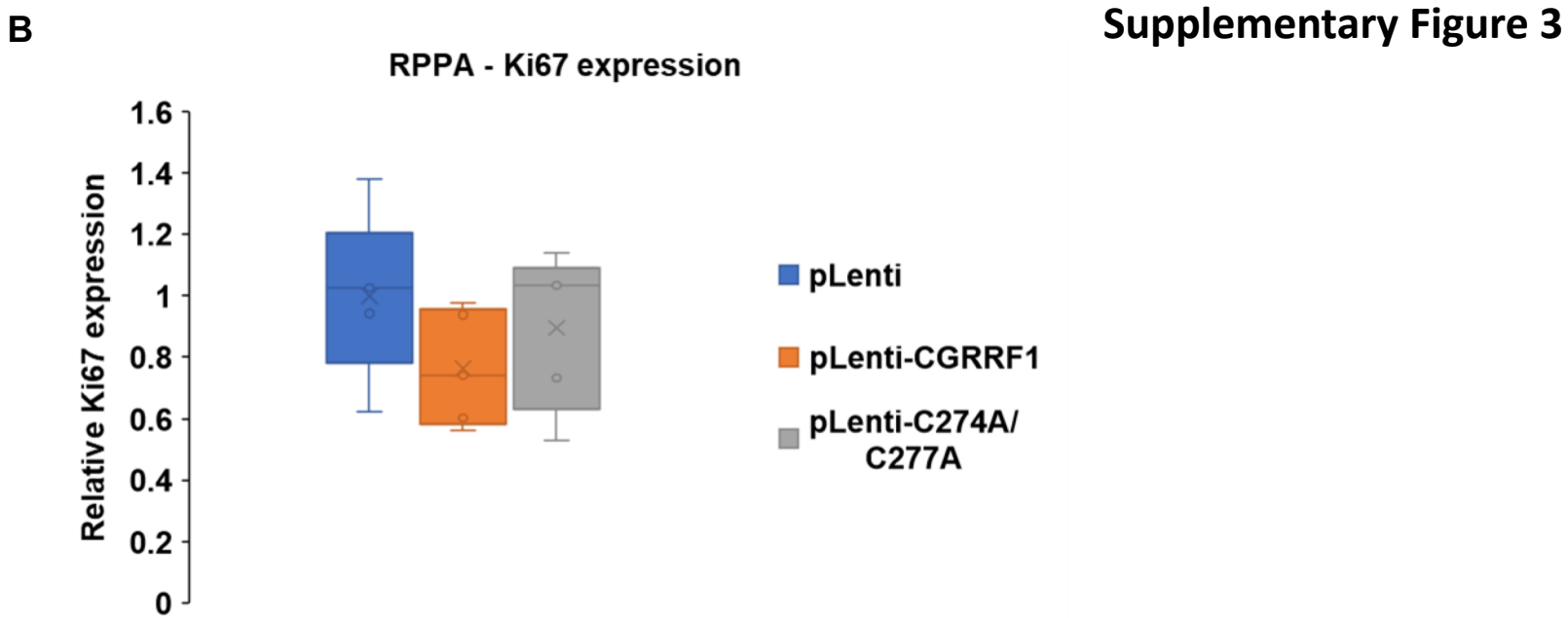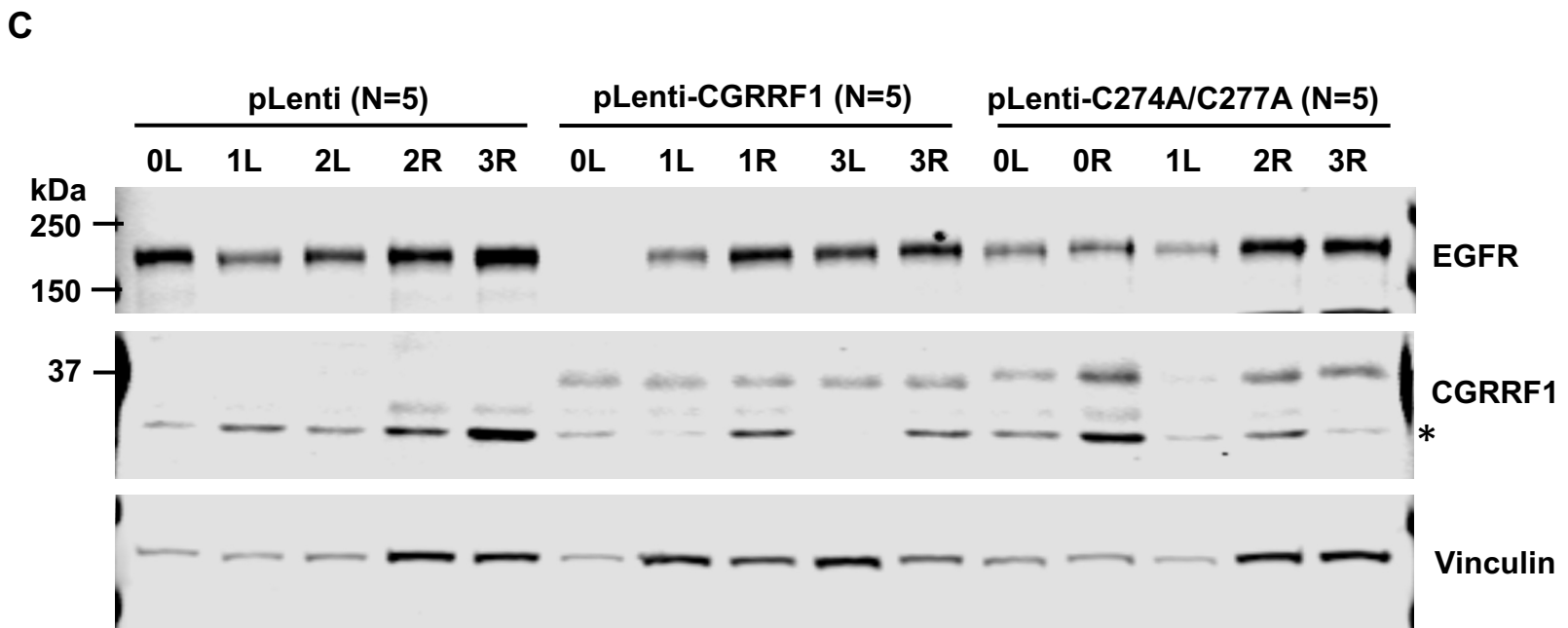

Supplement: Supplementary file 3 — Additional file 3: Figure S3. (A) The heatmap summarizes the RPPA profiling of the proteins which have significant (p < 0.05) difference among control, wild-type, and mutant CGRRF1 groups. (B) Ki67 level from RPPA analysis in each sample was normalized to the average of the pLenti group. Although the difference is not statistically significant, there is a trend of lower levels of Ki67 in wild-type CGRRF1 group compared with the other groups. (C) EGFR levels in xenograft lysates were examined by western blot analysis. Vinculin was used as a loading control. Nonspecific band is marked with an asterisk (*). [file 13058_2019_1212_MOESM3_ESM.pdf]

Supplementary Figure 4

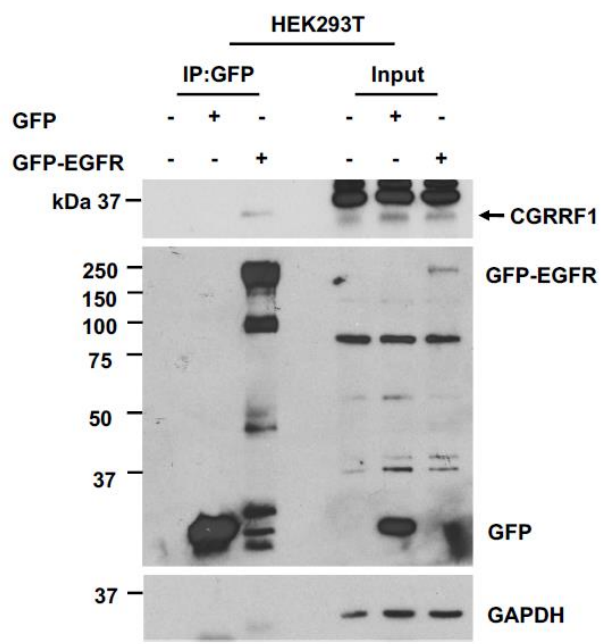

Supplement: Supplementary file 4 — Additional file 4: Figure S4. GFP-tagged EGFR interacts with endogenous CGRRF1. GFP or GFP-EGFR was transfected into HEK293T. 48 h after transfection, lysates were prepared and pulled down with anti-GFP beads. The bound CGRRF1 was detected by western blot using anti-CGRRF1 antibody. CGRRF1 signal was indicated by an arrow. [file 13058_2019_1212_MOESM4_ESM.pdf]

Supplementary Figure 5

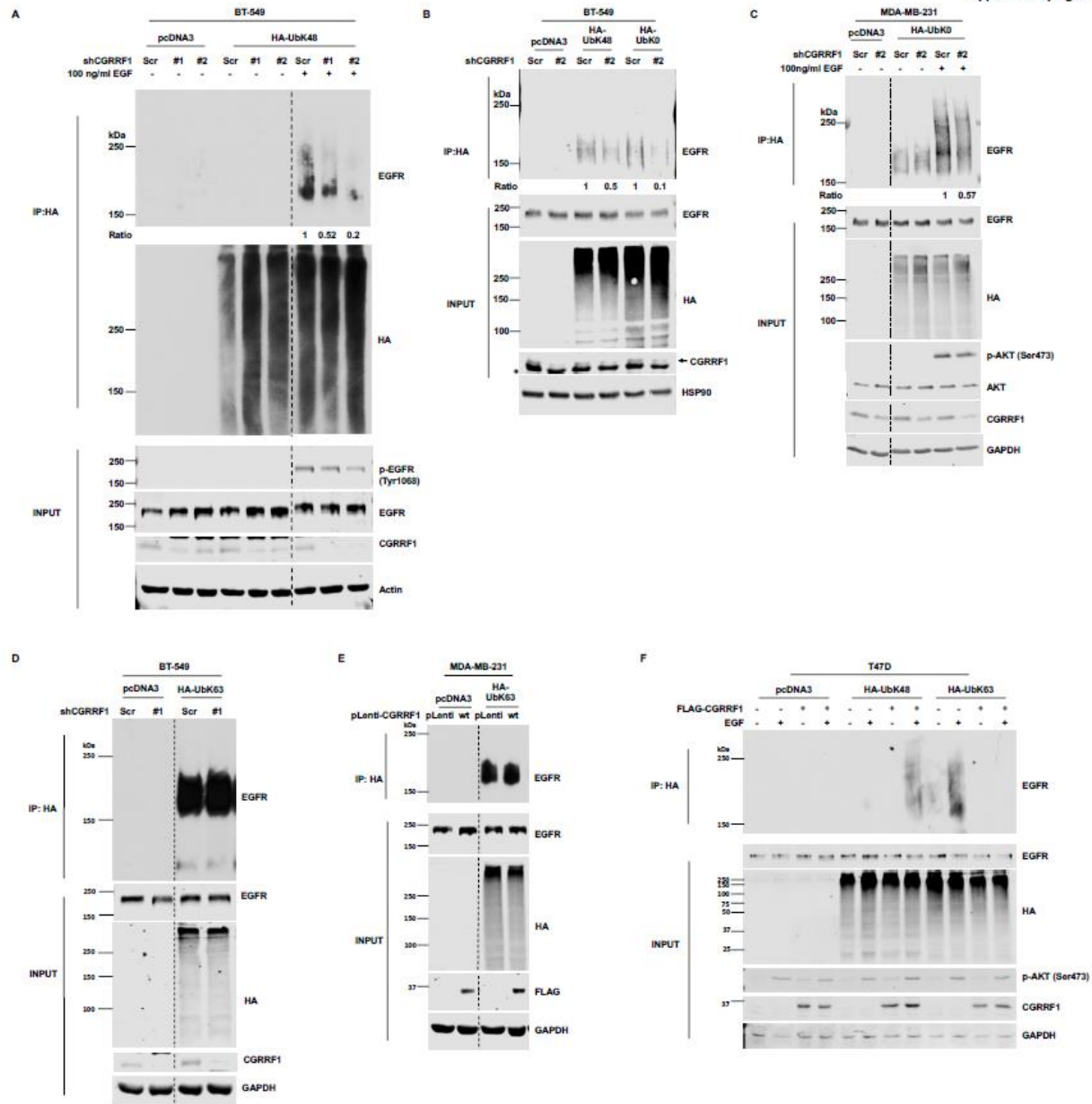

Supplement: Supplementary file 5 — Additional file 5: Figure S5. (A) CGRRF1-knockdown BT-549 cell lines were transfected with pcDNA3 or HA-UbK48. Next day, cells were serum-starved for 24 h, and then treated with 100 ng/ml EGF for 15 min. Cell lysates were harvested and in vivo ubiquitination assay was performed. (B) CGRRF1-knockdown BT-549 cell lines were transfected with pcDNA3, HA-UbK48, or HA-UbK0. Lysates were harvested 48 h after transfection and subjected to in vivo ubiquitination assay. CGRRF1 signal was indicated by an arrow, and a nonspecific band was marked with an asterisk (*). (C) CGRRF1-knockdown MDA-MB-231 cell lines were transfected with pcDNA3 or HA-UbK0. Next day, cells were serum-starved for 24 h, and then treated with 100 ng/ml EGF for 5 min. Cell lysates were harvested and in vivo ubiquitination assay was performed. (D) CGRRF1-knockdown BT-549 cell lines were transfected with pcDNA3 or HA-UbK63. Lysates were harvested 48 h after transfection and subjected to in vivo ubiquitination assay. (E) CGRRF1-overexpressing MDA-MB-231 cell lines were transfected with pcDNA3 or HA-UbK63. Lysates were harvested 48 h after transfection and subjected to in vivo ubiquitination assay. (F) T47D cells were co-transfected with FLAG-CGRRF1, HA-tagged UbK48 or UbK63. Next day, cells were serum-starved for 24 h, and then treated with 100 ng/ml EGF for 30 min. Lysates were harvested and followed by in vivo ubiquitination assay. [file 13058_2019_1212_MOESM5_ESM.pdf]

Supplementary Figure 6

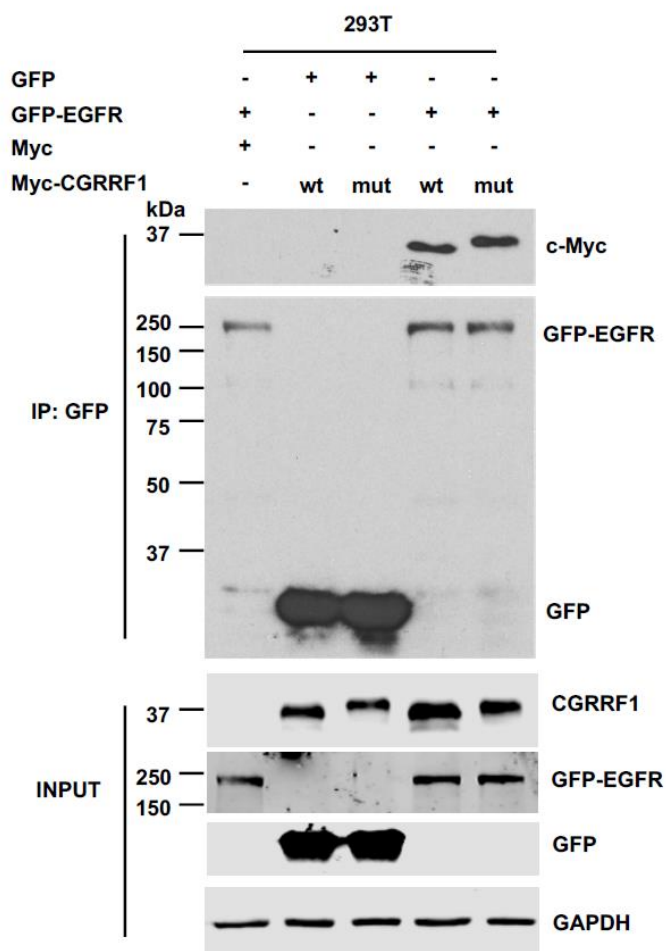

Supplement: Supplementary file 6 — Additional file 6: Figure S6. HEK293T cells were co-transfected with GFP-EGFR, Myc-tagged wild-type or mutant CGRRF1. Lysates were harvested 48 h after transfection, and GFP-EGFR was pulled down with GFP beads, followed by immunoblotting using indicated antibodies. [file 13058_2019_1212_MOESM6_ESM.pdf]

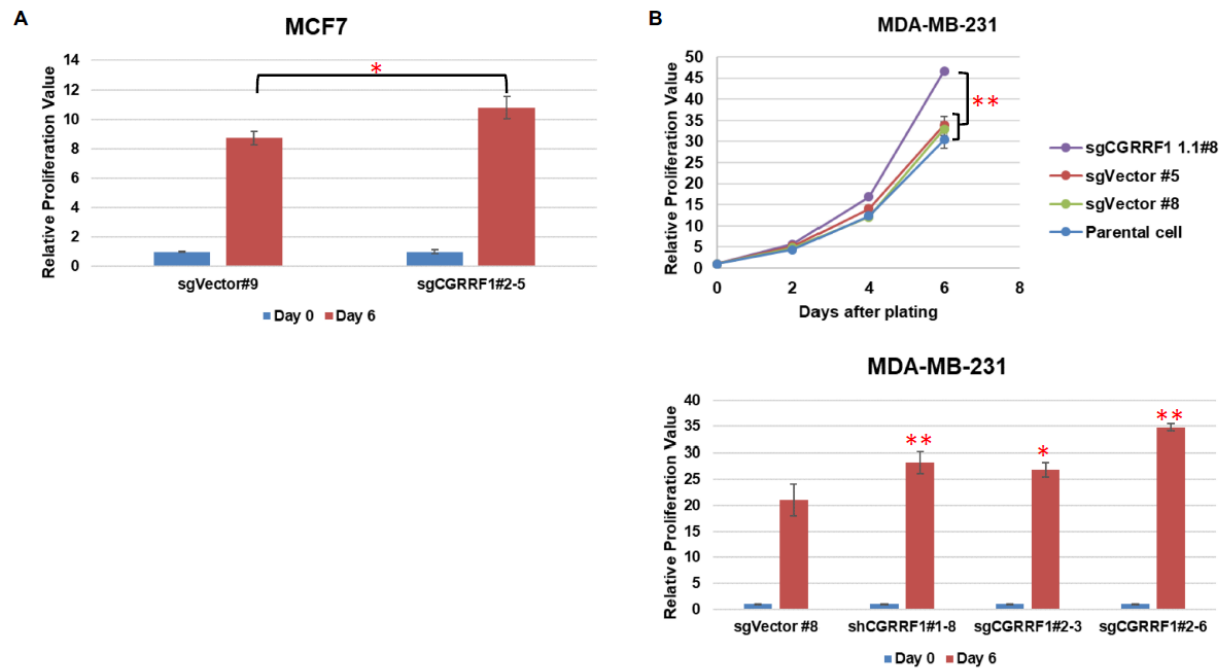

Supplementary Figure 7

Supplement: Supplementary file 7 — Additional file 7: Figure S7. Knockout of CGRRF1 enhances cell proliferation. (A) The growth rate of CGRRF1-knockout MCF7 cell line (sgCGRRF1#2–5) was determined by MTT assay. Error bars represent mean ± SD (n = 6). *p < 0.01. (B) The growth rate of CGRRF1-knockout MDA-MB-231 cell lines were determined by MTT assay. Error bars represent mean ± SD (n = 6). *p < 0.01, **p < 0.001. [file 13058_2019_1212_MOESM7_ESM.pdf]

## Supplementary Figure 8

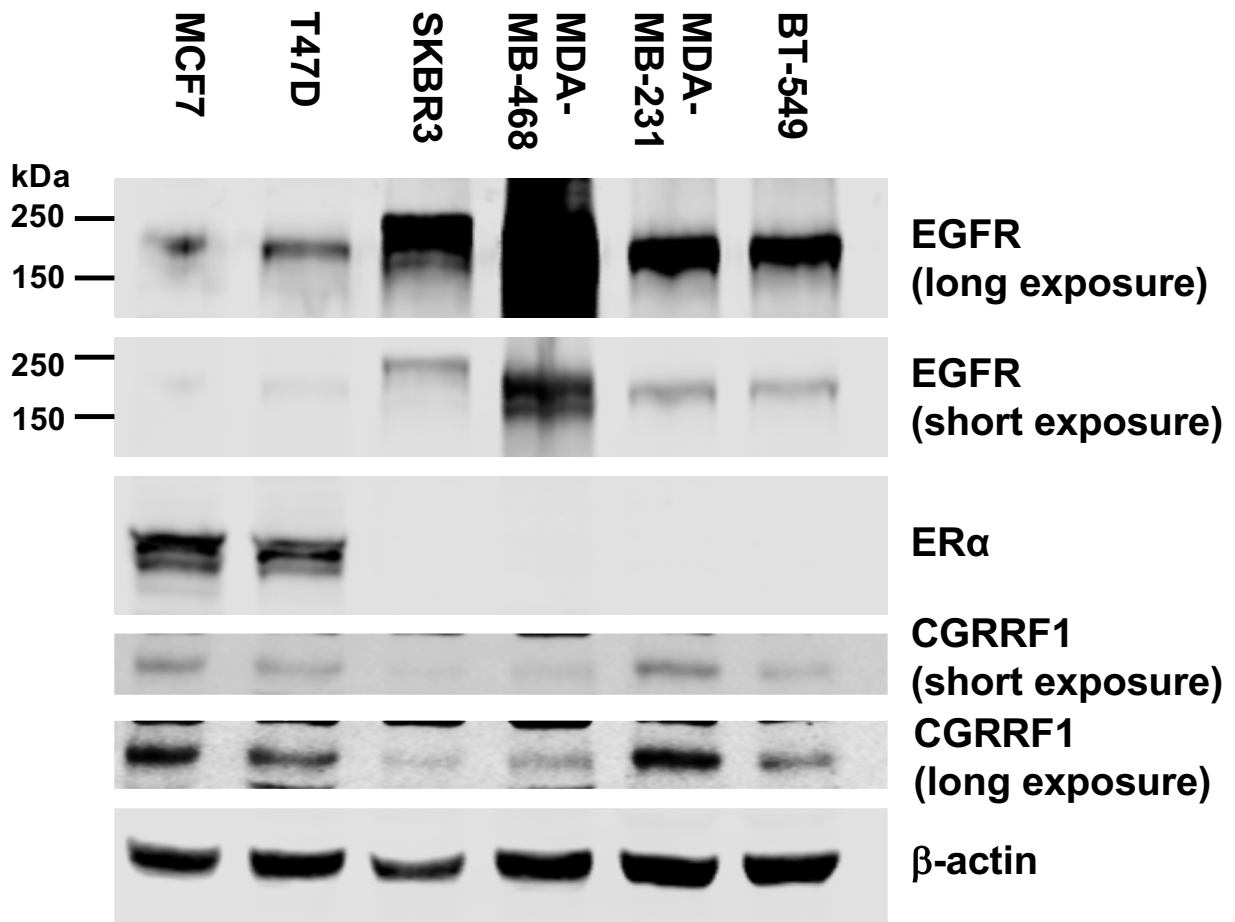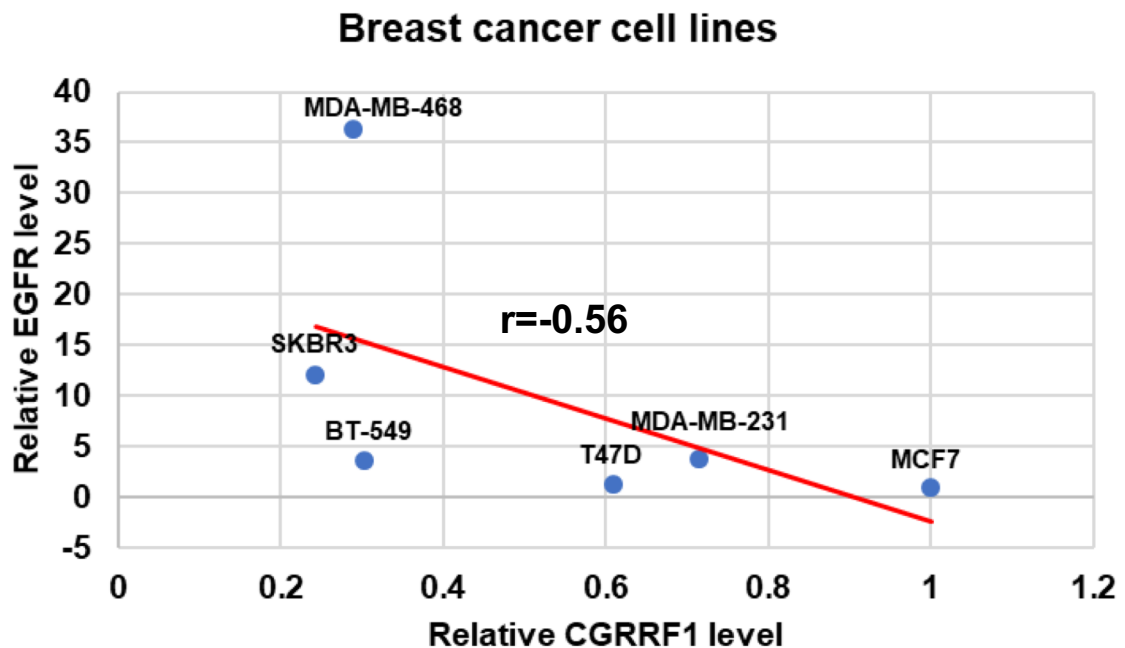

Supplement: Supplementary file 8 — Additional file 8: Figure S8. Correlation between CGRRF1 and EGFR protein levels in breast cancer cell lines. The membrane in Fig. 1A was probed with EGFR and the levels between CGRRF1 and EGFR were quantified by Infrared Imaging, normalized to that in MCF7 cells and then correlated. Pearson correlation coefficient R = -0.56. For easy readability and comparison, the western blots in Fig. 1A are shown again side-by-side with EGFR blots. [file 13058_2019_1212_MOESM8_ESM.pdf]

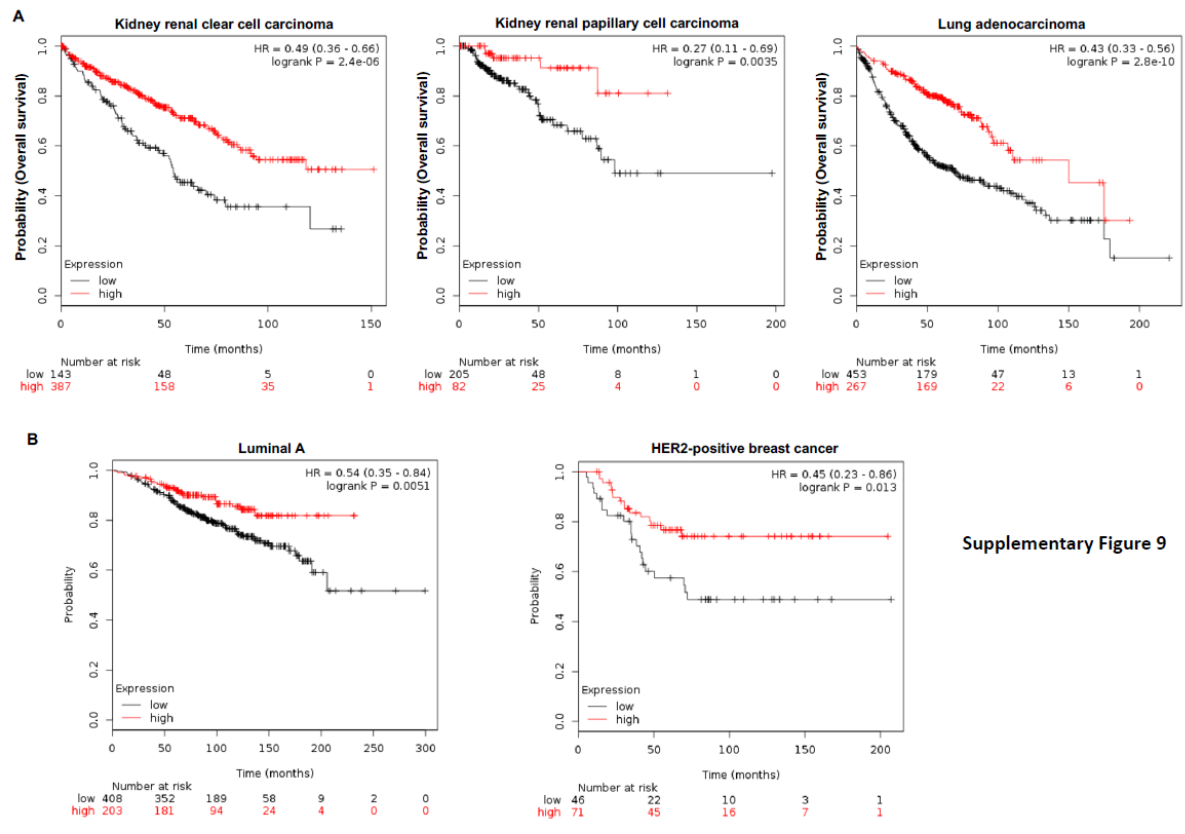

Supplementary Figure 9

Supplement: Supplementary file 9 — Additional file 9: Figure S9. Cancer patients with lower CGRRF1 had poor survival. (A) Kaplan-Meier curves of the overall survival of patients with kidney renal clear cell carcinoma, kidney renal papillary cell carcinoma, or lung adenocarcinoma. Patients were separated into two groups based on the expression of CGRRF1 in their tumors (data generated using KM Plotter server, kmplot.com, with auto select best cutoff and including all datasets in the server). (B) Kaplan-Meier curves in Luminal A and HER2-positive breast cancer patients. Patients were separated into two groups based on the expression of CGRRF1 (data generated using KM Plotter (auto select best cutoff, overall survival, and including all datasets in the server)). [file 13058_2019_1212_MOESM9_ESM.pdf]

A

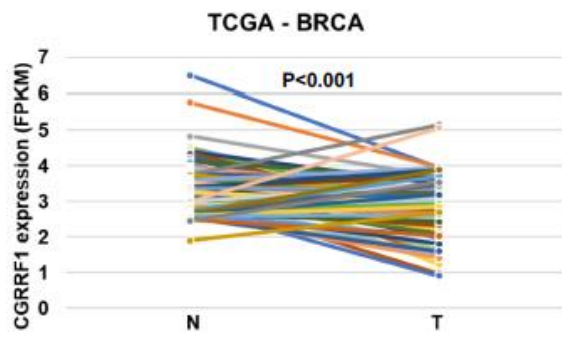

B

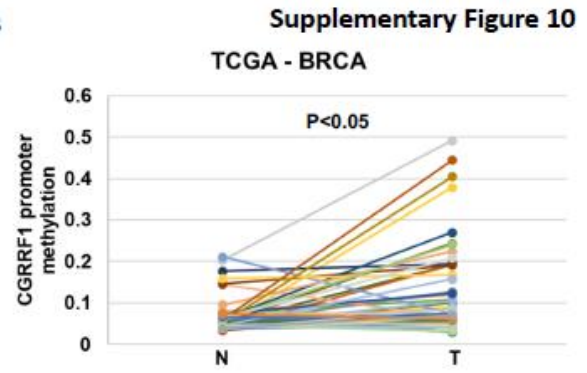

C

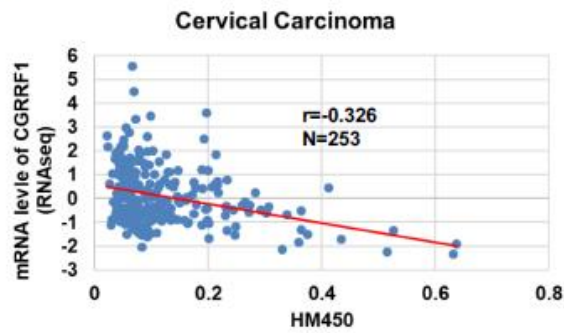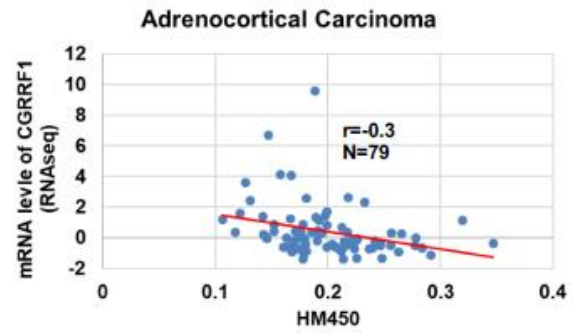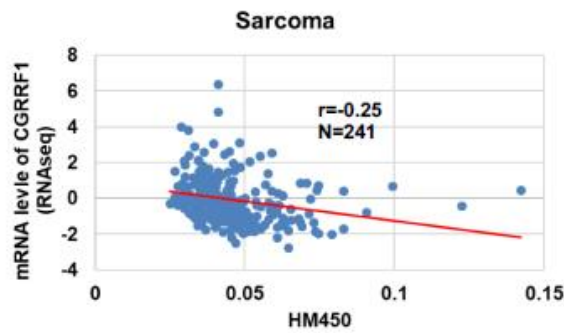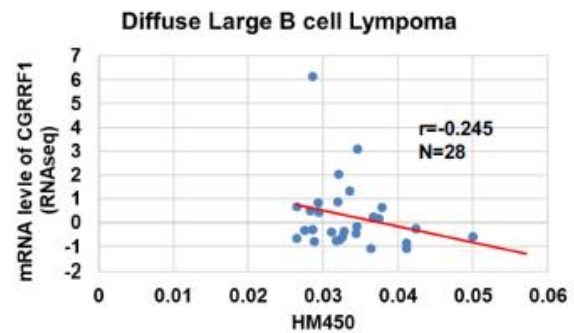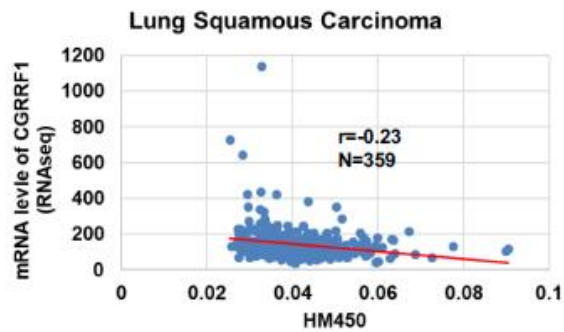

Supplement: Supplementary file 10 — Additional file 10: Figure S10. There is a negative correlation between the CGRRF1 mRNA expression and its promoter methylation status among different cancers. (A) CGRRF1 expression in normal breast tissues (N) and matched breast tumor samples (T) (data extracted from TCGAportal, TCGA BRCA, n = 76 pairs). (B) CGRRF1 promoter methylation status in patients with decreased CGRRF1 expression in tumor samples (data extracted from TCGAportal, TCGA BRCA, n = 57 pairs). (C) Pearson correlation coefficients between CGRRF1 mRNA levels and CGRRF1 promoter methylation (HM450) in different types of cancer (data extracted from cBioPortal, TCGA provisional). [file 13058_2019_1212_MOESM10_ESM.pdf]

**Supplementary Figure 11**

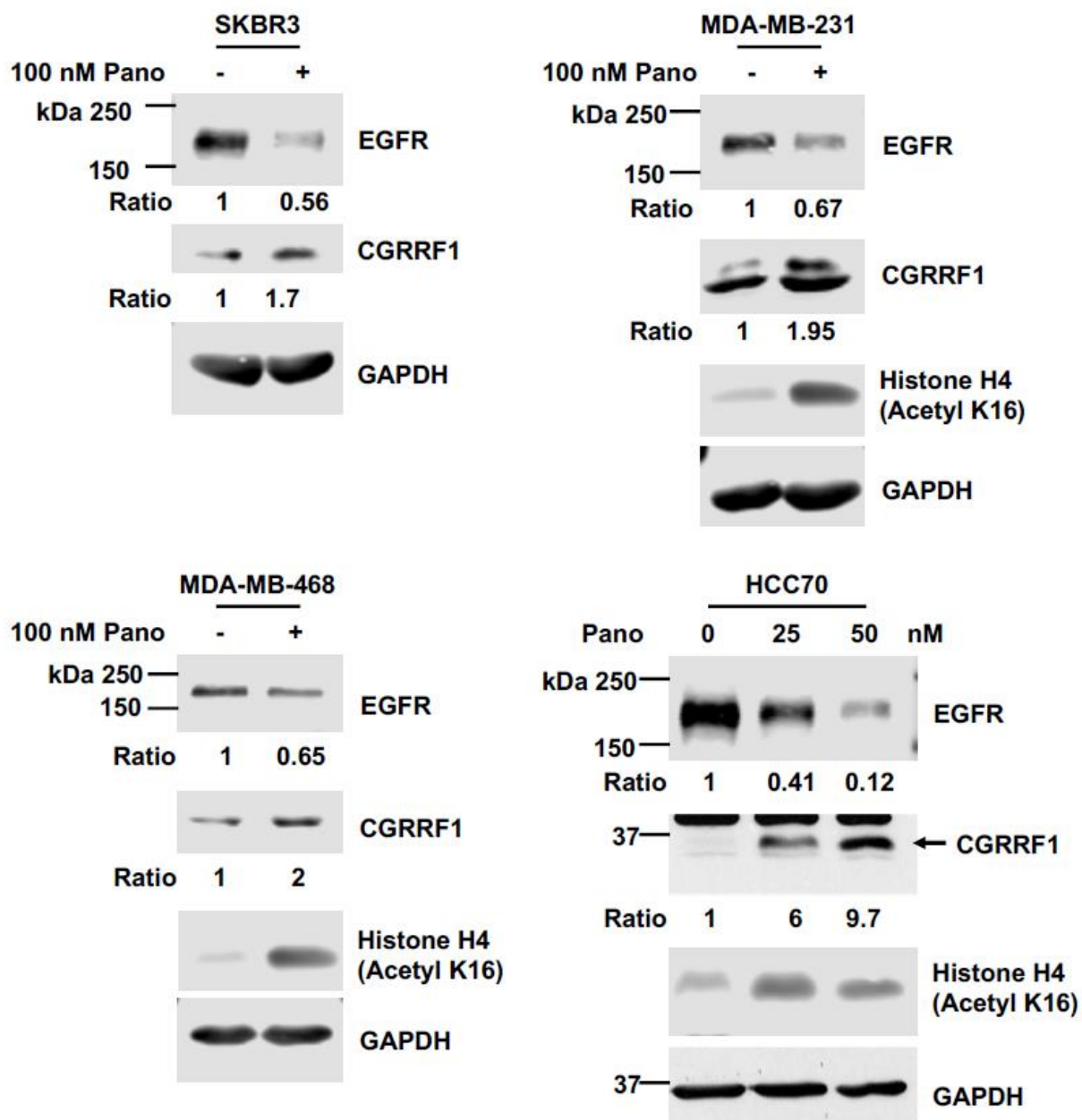

Supplement: Supplementary file 11 — Additional file 11: Figure S11. Panobinostat treatment increases the protein expression of CGRRF1 but reduces EGFR protein level. Cells were harvested 24 h after the treatment of panobinostat. The expression of CGRRF1 and EGFR was measured by western blot. Histone H4 K16 acetylation served as a positive control for panobinostat treatment. [file 13058_2019_1212_MOESM11_ESM.pdf]
